# The covalent docking software landscape: features and applications in drug design

**DOI:** 10.1093/bib/bbaf697

**Published:** 2025-12-31

**Authors:** Natesh Singh, Philippe Vayer, Bruno O Villoutreix

**Affiliations:** Evotec SE, Molecular Architects, Integrated Drug Discovery, Campus Curie, 195 Rte d'Espagne, 31100 Toulouse, France; Université Paris Cité, Inserm UMR 1141, Hopital Robert-Debré, 48 boulevard Sérurier, 75019 Paris, France; Université Paris Cité, Inserm UMR 1141, Hopital Robert-Debré, 48 boulevard Sérurier, 75019 Paris, France

**Keywords:** drug discovery, covalent docking, AI-powered drug design, ADMET

## Abstract

Covalent small-molecule ligands have re-emerged as powerful tools in drug discovery, offering prolonged target engagement, enhanced potency, and the ability to modulate proteins once considered undruggable. However, the rational design and virtual screening (VS) of covalent ligands remain challenging. Many docking tools cannot accurately model the energetics of covalent bond formation, often requiring more rigorous quantum mechanical (QM) or semi-empirical QM calculations for reliable predictions. Despite these limitations, the computational landscape is rapidly evolving. An increasing number of open-source, commercial, and web-based platforms now support binding mode exploration, lead optimization, and structure-based VS of covalent ligands. Alongside traditional approaches, new artificial intelligence (AI) and machine learning (ML) tools are assisting in prioritizing candidate molecules. This review introduces the fundamental principles and mechanisms of covalent inhibition, then provides a comprehensive overview of computational tools including covalent docking, warhead placement algorithms, and pharmacophore modeling, supporting early-stage drug discovery and chemical biology. Case studies highlight practical applications. We also cover curated databases of covalent binders and experimental 3D protein–ligand complexes, plus tools for assessing nucleophilic residue reactivity, all essential for robust covalent modeling. Finally, we briefly address risks associated with covalent chemistry. While progress is notable, further advances are needed. Nonetheless, today’s covalent docking and AI-driven tools already make a meaningful impact by enabling rational design, generating new ideas, refining hypotheses, and expanding the boundaries of druggability.

## Graphical Abstract

**Graphical Abstract:**
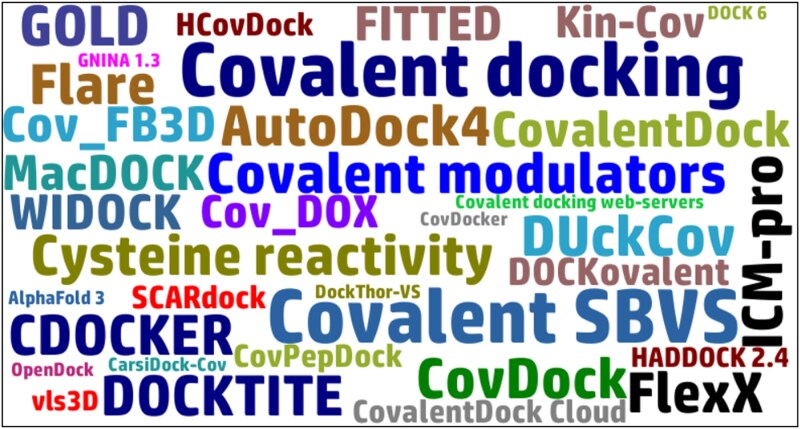

## Introduction

Over the past decades, the rational design of covalent ligands has gained traction and garnered increased interest [1–7]. Such molecules are often inhibitors, but can also act as activators or even be covalent allosteric modulators [8]. Unlike traditional noncovalent drugs that form reversible interactions with their targets, covalent ligands form chemical bonds with specific nucleophilic residues in proteins, leading to increased potency and the ability to target challenging binding sites. Studies have highlighted both the rewards and risks of covalent ligands [1, 9–11], and some of these risks involves nonspecific reactivity with proteins, DNA, or small molecules, which can cause acute or delayed toxicity [12–14], even in some cases triggering immune responses [15]. Despite these concerns, covalent ligands demonstrate stronger potency, prolonged target engagement, enhanced selectivity, and effectiveness against resistant mutants compared to reversible inhibitors [1, 16]. Based on the analysis made by Dalton et al., 128 small molecule drugs act via a covalent mechanism, which covers ~7% of all FDA-approved drugs [17].

**Figure 1:**
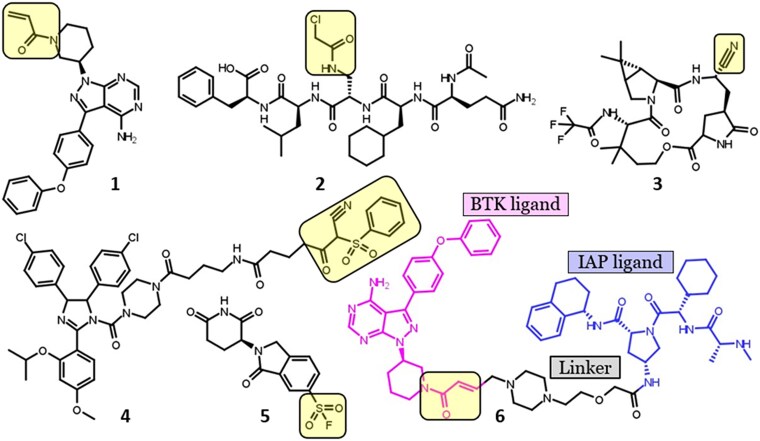
Examples of covalent ligands: **1**. Ibrutinib [21], **2**. Peptide-based covalent inhibitor of bacterial sliding clamp [22], **3**. Nirmatrelvir-based macrocyclic covalent inhibitor of SARS-CoV-2 3CL^pro^ [23], **4**. Covalent inhibitor of HDM2/p53 PPI [24], **5**. covalent inhibitor of the cereblon E3 ubiquitin ligase complex [25], **6**. Ibrutinib-based BTK-cIAP1-Covalent degrader [26]. The warhead on the ligand is highlighted in the yellow slab.

Historically, many covalent drugs were discovered serendipitously, such as aspirin (marketed over 100 years ago) and penicillin (discovered in 1928) [6]. Today, rational strategies dominate, and reversible ligands can be designed into covalent binders by incorporating reactive warheads. For example, the BTK (Bruton’s Tyrosine Kinase) covalent inhibitor Ibrutinib (Fig. 1) (IC_50_ of 0.72 nM) was derived from a noncovalent ligand (IC_50_ of 8.2 nM) by adding a Michael acceptor electrophile. Beyond small molecules, covalent ligands now include peptides, macrocycles, protein–protein interaction (PPI) modulators, E3 ligase targeting covalent immunomodulatory imide drugs (IMiD’s), some molecular glues, and PROTACs (proteolysis-targeting chimeras), thus molecules that often bypass the rule of 5 [18] (Fig. 1). Also, in terms of targeted modalities, covalent binders are no longer limited to proteins only. Many recent studies have shown the development of covalent binders targeting RNA [19, 20], suggesting the broad applicability of bioactive compounds acting via covalent mechanisms.

**Figure 2:**
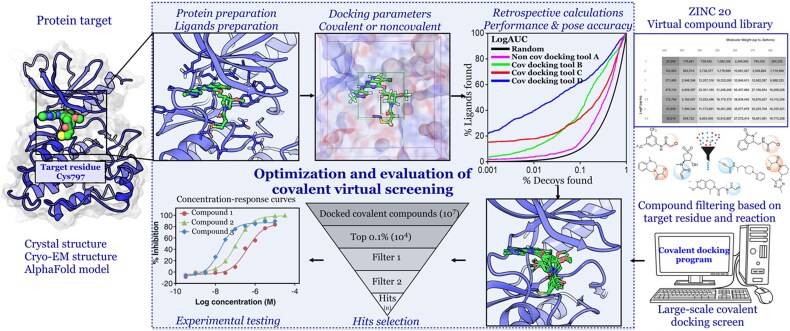
Covalent screening workflow. The two required inputs for such a screen are the target structure with a nucleophilic residue in the binding site and a covalent screening collection. The target structure is prepared and converted into the representation required by the docking software. This is followed by benchmarking the ability of different noncovalent and covalent docking programs using a set of active and inactive covalent compounds to correctly reproduce and score the docking poses if available. Optimization of the workflow requires the exploration of different docking parameters, such as grid size, specialized treatment of macrocycles or highly flexible ligands, side chain flexibility, torsional constraints, receptor desolvation, vdW scaling, number of poses sampled, postdocking minimization, rescoring, etc., depending on the docking engine(s) used. The best docking protocol or program is then used for prospective covalent VS. After docking, the top-ranked hits can be filtered and selected for experimental assays.

Experimental and computational methods jointly drive covalent drug discovery. Experimental methods include: Intact mass spectrometry (MS) screening [27]; Covalent fragment-based screening [28–30]; Isotopic tandem orthogonal proteolysis-activity-based protein profiling (isoTOP-ABPP) [31, 32]; and Covalent DNA-Encoded Library (DEL) screening [33, 34]. Among the computational strategies, structure-based virtual screening (SBVS) remains a popular approach (Fig. 2). While SBVS is usually efficient for noncovalent hits, the approach is not without pitfalls [35–40]. For instance, an important problem with SBVS (and ligand-based VS) is that the methods are usually target-dependent, and as such, one method cannot fit all [35, 37–39, 41, 42]. And there are even more challenges for covalent screening as the approach has to model the formation of a chemical bond while also considering the traditional noncovalent interactions that govern the initial binding event. Multiple covalent docking tools now try to address these challenges, employing varied strategies for sampling conformations, handling flexibility, and scoring ligand fits. Next to docking, other techniques (based on the 2D and the 3D knowledge of known ligands) such as shape screening, chemical fingerprint similarity [43, 44], electrostatic matching [45–50], pharmacophore modeling [51–53], quantitative structure–activity relationship (QSAR) [54–57], and artificial intelligence and machine learning (AI/ML) models [43, 44, 58–60] can be applied. But a full survey of these approaches lies beyond this article’s scope.

This review focuses on computational tools and web servers used to identify or optimize covalent ligands through covalent docking, VS, pharmacophore modeling, and reactive residue prediction. Where available, we highlight experimental case studies. The computer engines and workflows that we report are essentially peer-reviewed published methods with URLs tested up to November 2025 (Table S1) and most of them are also available online at www.vls3d.com [61–63]. Relevant ligand-protein databases about covalent binders are reported in the Supplementary Information. These resources were identified by internet and AI-assisted search, i.e. in addition to conventional literature search, we have also used ChatGPT’s ‘Deep Research’ capability (OpenAI, 2025), Gemini’s ‘Deep Research’ utility (Google, 2025) and Claude Sonnet 4.5 (Anthropic, 2025) to search for additional studies that may not have been consolidated yet in traditional academic databases. All insights derived from these AI-powered searches were then independently verified by the authors for accuracy and scientific relevance before inclusion. We believe that such hybrid approach balances human expertise with the efficiency of AI-augmented research.

### Covalent ligands: key concepts and tools

The binding of a targeted covalent ligand to a protein generally follows a two-step mechanism: (i) formation of a noncovalent complex driven by electrostatics, hydrogen bonding, hydrophobic, and van der Waals interactions, and (ii) covalent bond formation between the ligand’s electrophilic warhead and the target’s nucleophilic residue. This two-step mechanism and associated kinetic determinants are discussed in the Supplementary Information and Fig. S1. While cysteine is the most common target due to its high reactivity, warheads have also been designed for serine, lysine, and histidine [64–69].

**Figure 3:**
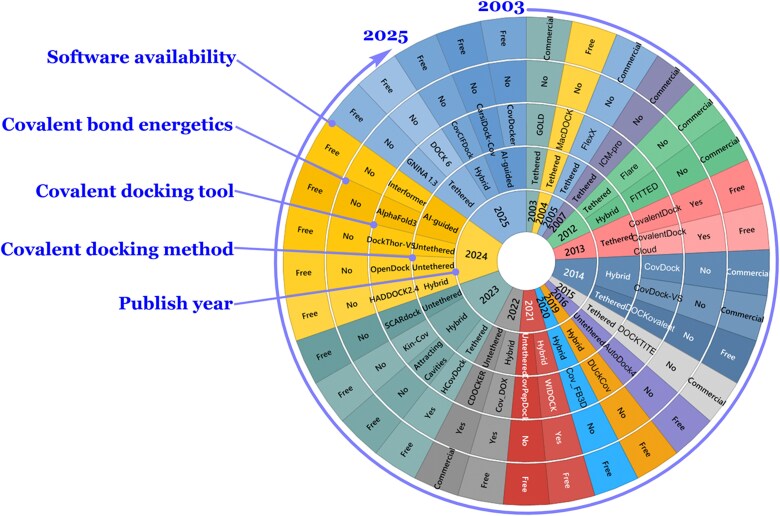
The covalent docking software landscape is shown as a sunburst chart. The landscape demonstrates the evolution of covalent docking tools, where an early development of tools based on the tethered approach can be observed, followed by the emergence of some tools based on the untethered approach. The landscape is then largely dominated by hybrid approaches, followed by the recent development of AI-guided covalent docking tools. Based on this trend, it seems reasonable to suggest that future tools based on improved DL algorithms or a combination of AI and physics-based approaches would likely emerge to further improve the accuracy of predictions. Due to the rapid pace of new tool development, the figure is not intended to be fully exhaustive. Emerging methods are discussed in the main text.

Covalent pose prediction tools can be broadly grouped into four categories: (i) **Tethered** or direct linking approaches: the covalent complex is generated based on the known chemistry and geometry of the covalent bond between the warhead and the residue. Subsequently, the docking engine is used to optimize the ligand conformation, followed by the assessment of noncovalent interactions. Since translational degrees of freedom are ignored, this method is computationally efficient and commonly used. Ligands are typically supplied in a postreacted state unless converted automatically by the software. (ii) **Biased** or constrained approaches: Here, the ligand and target attachment sites are modified, and forcefield constraints or potentials guide the warhead near the reactive residue, typically using distance or pharmacophoric restraints. Ligands are docked in a nonreacted state. Both tethered and biased approaches assume a fixed covalent geometry and mainly score poses based on noncovalent interactions [70]. (iii) **Hybrid** approaches: Combining tethered docking with advanced sampling techniques like simulated annealing (SA), metadynamics, or molecular dynamics (MDs), these methods are more complex and computationally intensive. They often mimic the two-step binding mechanism by first assessing noncovalent affinity and then estimating reaction probability based on warhead proximity and orientation. Some tools also incorporate covalent bond energetics using molecular mechanics (MMs) or quantum calculations (QMs and semi-empirical QM) [70]. In these methods, compounds are usually provided in a nonreacting state, which are later modified to form covalent complexes once reasonable poses are found. (iv) **AI-based** tools: Emerging deep-learning approaches can prioritize reactive residues or generate ligands de novo using only binding pocket information. Some integrate AI-based scoring or co-folding predictions. However, most current AI-guided tools neglect covalent bond energetics and require postprocessing to validate poses and assess chemical feasibility. Often, these AI tools have not been fully developed for covalent docking but have the potential of being used for such endeavor assuming additional tuning. The covalent docking software landscape is shown in Fig. 3. Whereas, a detailed information on different covalent docking programs, web servers, and pharmacophore modeling tools with URLs is provided in Table S1.

### Tools based on the tethered approach

#### GOLD

GOLD is an automated commercial docking tool based on genetic algorithms (GA) [71–73]. Its covalent docking methodology relies on the superposition of a ‘link atom’ in both the ligand and protein structures to utilize postdocking ligand conformations. Manual modification of the input files is required to bind the reactive sulfur or oxygen atom type of the targeted amino acid residue (Cys, Ser, or Thr) to the ligand’s electrophilic atom. The ligand’s link atom is forced to fit onto the protein’s link atom, and the correct geometry of the bond between ligand and protein is ensured with an angle-bending potential from the Tripos Force Field [74]. The GOLD Fitness Score is used to rank the ligand docking conformations, with the best pose receiving the highest score. The energy of covalent bond formation is not considered in the scoring functions of GOLD. Recently, Xu et al. used GOLD for performing structure-based optimization of a class of acrylamide-based menin inhibitors, which led to the discovery of M-808 as a highly potent and efficacious covalent menin inhibitor with the affinity of 1 and 4 nM, respectively, in inhibition of cell growth in the MV4;11 and MOLM13 cell lines carrying MLL fusion. In another study, Endres et al., by combining the noncovalent docking of GOLD and MD simulations against 20 complexes of cathepsin K, a papain-like cysteine protease, showed that the noncovalent docking approach can be useful for modeling of covalent ligands [75].

#### MacDOCK

MacDOCK is a hybrid program for similarity-driven docking calculations [76] (with incremental construction algorithm and grid-based scoring) based on DOCK 4.0 [77, 78] and the superposition program MIMIC [79]. It is the first docking program i.e. specifically adapted for performing VS of covalent ligands [76, 80]. For covalent ligands, an anchor-guided docking is used, in which information about the location and directionality of the atoms involved in covalent binding is incorporated to guide the placement of the ligand (through similarity calculations) towards an orientation compatible with the covalent bond formed between ligand and protein. Before docking, functional groups in the ligand and the site in the protein forming the bond must be identified and, if necessary, transformed, so that the arrangement of all the atoms matches that of the product structure.

#### FlexX

FlexX [81–83] is a commercial software for performing covalent docking developed by BioSolveIT GmbH. The FlexX performs covalent docking through a direct linking approach where the covalent link between the ligand and the receptor is defined manually. On the receptor side, the ligand structure input file is extended to include the two atoms nearest to the covalent linkage. The first placement is accomplished by superimposing these atoms on their receptor structure sites. According to a stepwise evaluation by the software’s empirical scoring function, the standard incremental construction method results in the ultimate placement of the remaining parts of the ligand in the most suitable parts of the pocket. The recent version of SeeSAR (v. 11.2), a 3D desktop modeling platform of BioSolveIT, is accompanied by a workflow called ‘CovXplorer’ for performing covalent SBVS. The CovXplorer platform performs covalent screening in a more hybrid manner than the conventional FlexX program. In the first step, the ligand is docked noncovalently in the binding site where the electrophilic warhead is placed near the targeted residue. After the generation of docking poses, they are assessed for their binding affinity, and promising candidates are transferred to the next stage. Here, warheads are processed and translated into their ligand-binding form. In the final step, the processed compounds are docked covalently and once again assessed for their binding affinity with HYDE [84–86] to allow ranking and visual inspection of the results to select compounds for a follow-up.

#### ICM-Pro

ICM-Pro is a commercially available docking program developed by Molsoft LLC [87]. ICM-Pro automatically turns the prereaction ligand into a ‘pseudo-ligand’ based on the reaction type by covalently connecting the cysteine side chain to the electrophilic warhead in all stereoisomers formed upon addition. The protein’s sidechain atom coordinates are used to constrain the placement of the pseudo-ligand and then deleted to avoid unfavorable collisions during docking. Monte Carlo simulations are used to sample ligand conformations in a set of pocket-specific grid maps [88]. Finally, a modified version of the usual ICM-Pro scoring function is utilized to evaluate docking conformations by disregarding pairwise interactions between atoms immediately connected to the new covalent bond. The energy of covalent bond formation is not considered in the scoring function.

Katritch et al. used ICM-pro for performing covalent VS using a large covalent database of ~230,000 molecules against the homology model of I7L ubiquitin-like proteinase (ULP) of the vaccinia virus [89]. The best compounds showed IC_50_ values of <6.2, 14.19, and 195 μM [2, 89].

#### Flare

Flare [90] is a commercial docking software from the Cresset group that can perform covalent docking using the Lead Finder (LF) docking algorithm [91–93]. To run a covalent docking, the ligand to be docked should be provided in an appropriate reactive electrophilic form, and the nucleophilic amino acid in the active site must be specified before docking. The workflow then performs a chemical transformation to the input compound, which modifies the covalent warhead to generate a pseudo-covalent ligand. This is not formally covalently bonded to the protein but is bonded to the nucleophilic atom from the selected amino acid, which is protonated. This is followed by docking of the ligand to the nucleophilic residue to explore different poses where the ligand is bonded to the nucleophilic atom of the selected amino acid, which is protonated. Finally, the results are postprocessed to select the best candidates based on scoring values. LF scoring functions are based on a semi-empirical MM function that explicitly accounts for various types of molecular interactions [92].

#### CovalentDock

CovalentDock is a covalent docking package developed by Ouyang et al. [94] which is based on AutoDock. In this docking tool, an empirical model of free energy change was developed for the estimation of covalent linkage formation while handling the molecular geometry constraints of the covalent linkage with special atom types and directional grid maps. The interaction between the ligand and its receptor is modeled like any conventional docking program through noncovalent interactions such as van der Waals, electrostatics, solvent effects, etc. [94].

#### DOCKTITE

It is the SVL implementation of a highly versatile workflow for covalent docking, developed by Scholz et al., in the Molecular Operating Environment (MOE) [95]. Briefly, the different steps of covalent docking using DOCKTITE involve: (i) Warhead Screening*—*a database of ligands is searched for electrophilic warheads. Subsequently, the electrophilic atom is tagged and the ligand is converted to its bound shape; (ii) Side chain Attachment—the tagged ligand atom is now attached to the nucleophilic side chain of the receptor, and for prochiral warheads, stereoisomers of the chimeric molecules are generated; (iii) Pharmacophore-guided docking – The program generates an automatic pharmacophore model for the attached residue and analyzes the active site automatically. This model is used for an exact positioning of the nucleophilic side chain during the docking step; and (iv) Side chain Cleavage & Pose Rescoring—this step estimates the final docking scores of the poses after being disconnected from their attached side chain. After the fully automated cleavage step, the rescoring is realized by a consensus scoring approach using MOE-internal scoring functions (Affinity dG or London dG) and the external knowledge-based scoring function DSX [96]. The energy of covalent bond formation is not considered in the scoring functions.

#### GNINA 1.3

GNINA is an open-source molecular docking software, a fork of Autodock Vina [97, 98] and Smina [99]. In the first step, the ligand conformational sampling is carried out via a set of Markov chain Monte Carlo (MCMC) chains that randomly perturb the ligand in the specified binding site [100, 101]. Following sampling, top poses are retrieved based on the scoring of protein-ligand conformations. Gnina utilizes convolutional neural network (CNN) scoring functions that work on an atomic density grid representation (i.e. a 3D ‘picture’ of the complex) within the docking workflow [102]. The ligand poses from the MCMC chains are first minimized with respect to the Autodock Vina scoring function, and then rescored and ranked using the CNN scoring functions. An ensemble of CNN scoring functions of differing computational complexity is used to score the ligand poses, which enhances the binding pose prediction at the cost of additional computation. The GNINA v. 1.3 also provides an interface for covalent docking. The input ligand must be provided as a conformation representative of the bound form. Additional optional arguments to refine the positions and treatment of the covalent bond can also be specified.

#### DOCK 6

DOCK 6 [103] is the latest version of DOCK [104], which is a molecular modeling program to identify potential binding geometries and interactions of a molecule to a target. In DOCK 6, the authors have implemented receptor desolvation scoring functions and tested two solvation methods that account for receptor desolvation within the scoring functions: Grid-based IST (GIST) and 3D-RISM [105, 106]. Importantly, DOCK 6 comes with a covalent docking algorithm based on an attach-and-grow principle. In this method, the molecule is first attached to the receptor at the specified covalent residue, and then the molecule is grown out to generate the pose. That is, user-specified covalent bond information is used to position the attachment point, then the molecule is revealed one segment at a time, and degrees of freedom are sampled until the molecule is fully displayed.

### Tools based on the untethered approach

#### AutoDock4

AutoDock4 (AD4) is an open-source docking tool developed at the Scripps Research Institute [107]. There are two kinds of covalent-docking methods supported in AD4: the two-point attractor method (untethered docking) and the flexible side chain method [107, 108] (tethered docking). Both methods utilize grid maps that have been precalculated with atom probes to accelerate the scoring process, but they differ in how they mimic ligand conformations during the scoring process. The two-point attractor approach first clips the target residue side chain to remove two terminal atoms (i.e. Cβ and Sγ for cysteine). These two atoms are then attached to the alkylating ligand at the appropriate location with ideal chemical geometry and assigned two special atom types (X and Z). Two specialized interaction maps are then created for these atom types, with a Gaussian potential (termed as Z-potential) centered on the location of the original atoms in the receptor structure, with a negative value close to the desired location and rising to zero at distant locations. The Z-potential penalizes the poses when Z or X atoms are outside their covalently attached location, thus driving the ligand into a proper pose. ​ In the flexible side chain method, a ligand coordinate file is modified by connecting the two target residue atoms at the site of alkylation, with ideal chemical geometry. These two ligand atoms are then overlapped with the matching atoms in the receptor structure to establish the covalent bond with the residue before running the docking. Then, during the docking, the complex is treated as a fully flexible side chain, using the conventional AD4 technique for modeling residue flexibility. AutoDock Vina, a variant of AutoDock, is one of the fastest and most widely used open-source docking engines that can be used to model covalent docking [97, 98]. However, the option for modified energy evaluation is not available in Vina. In a recent work, Bianco et al. presented a modified reactive docking protocol based on AD4, where they adapted AutoDock’s standard force field [108], which in turn modifies the near attack conformation [109] (NAC), the last ground state geometry preceding the transition state geometries, with a decreased equilibrium distance for the reactive atoms. With this model, the ligand is docked in the unmodified state before the reaction, with the reactive warhead still intact. Another advantage of using this approach is that no expensive QM calculations are required to determine the ligand and receptor geometries [110].

#### CDOCKER

CDOCKER is an implementation of covalent docking in Rigid CDOCKER, a grid-based MD docking algorithm, by Wu et al, where a customizable grid potential has been introduced to mimic the free energy change for the chemical reaction that forms the covalent bond [111]. To overcome the limitation of the two-point attractor method of AutoDock, which treats different bond formation reaction types with the same grid potential, the authors have performed docking experiments on the pose prediction dataset using different covalent bond grid potentials to identify the best parameters for different reaction types.

#### OpenDock

OpenDock is an open-source, PyTorch-based protein-ligand docking framework designed for flexible protocol building and method development [112]. In this framework, the sampling and scoring modules are defined in a separate, objective-oriented manner, allowing users to build their own sampling and scoring methods. Several conformation sampling strategies, such as the Monte Carlo method (MC) [113], GA [114], and particle swarm optimization (PSO) [115], have been included within OpenDock. For improved performance in local optimization of docking poses, optimization methods such as Limited-memory Broyden-Fletcher-Goldfarb-Shanno (L-BFGS) [116], Adam [117], and stochastic gradient descent (SGD) [118] are included. Moreover, both the original AutoDock Vina scoring function and the deep learning (DL)-based scoring function DeepRMSD [119] are implemented for both global sampling and local optimization and external scoring methods [120, 121], and user-defined scoring functions can also be utilized in the docking process. The program also allows for setting up various constraints (such as distance) to support covalent docking and enzyme-substrate modeling.

### Tools based on the hybrid approach

#### Flexibility induced through targeted evolutionary description

Flexibility induced through targeted evolutionary description (FITTED) [122–124] is a fully automated commercial docking program of Molecular Forecaster [125] i.e. based on a GA. In FITTED, a covalent bond is not formed by a priori linking of the corresponding atoms. Instead, docking is carried out in the usual noncovalent form, and only the protein nucleophile is specified beforehand. The type of electrophilic group in the ligands of the library to be docked is identified automatically. If such a group is detected, the distance between the electrophilic atom and the protein nucleophile is monitored: if it is below a certain threshold (e.g. ideal covalent bond length plus 1 Å), the bond is formed. If the distance is larger, the ligand is maintained in its noncovalent binding mode. The covalent bond formation is not separately scored, which precludes an automated ranking of ligands with different warheads, and hence requires visual inspection of the final results as well as consideration of relative reactivities as derived, e.g. from QM calculations [126]. In a recent study, Plescia et al used FITTED for performing docking of a set of covalent inhibitors of prolyl oligopeptidase (POP) and fibroblast activation protein α (FAP) covalent inhibitors to investigate the influence of different reactive groups on both potency and binding kinetics on these two serine proteases [127].

#### CovDock

It is a commercially available tool developed by Zhu et al. [128] and is based on the Schrödinger Glide docking algorithm [129, 130] and Prime structure refinement methodology [131]. CovDock performs docking in a hybrid manner and has several stages: (i) The compounds are first docked with Glide in a noncovalent manner to identify bound poses that are suitable for covalent bond formation. To allow for different possible conformations of the side chains of the reactive residue, it is mutated to alanine in this stage; (ii) The next step is the covalent bond formation between the receptor and the ligand. In this stage, the reactive residue side chain is restored, and its rotamers are sampled to find the best conformation for each ligand pose. The poses are then minimized to relieve the strain induced by covalent bond formation; (iii) Poses are then clustered and a representative pose from each cluster is selected, followed by their full minimization with Prime. The poses for each ligand are then ranked by the Prime energy; and (iv) Poses are finally scored for the affinity of the ligand to the receptor for noncovalent binding prior to reaction. In this stage, the bond is broken again, the reactive receptor residue is mutated to alanine, and the bond to the ligand is capped with hydrogen. Scoring is then done with Glide, and the affinity is reported as the average of the prereacted and postreacted GlideScore for a given pose, as the property cdock affinity. In a recent study, Zhou et al. applied a hybrid strategy for covalent VS by combining warhead screening and preprocessing with GOLD and CovDock software using a ZINC virtual library. The best compound with a α-ketoamide warhead showed an IC_50_ of 12.4 μM [132] in the Proteasome inhibition assay.

#### CovDock-VS

CovDock-VS is an advancement of the CovDock tool developed by Warshaviak et al [133]. This VS protocol is at least 10 times faster than the default (‘pose prediction’) CovDock protocol and is suitable for screening thousands of ligands. The CovDock-VS protocol skips time-consuming steps such as rotamer sampling and minimization. Also, the affinity score and the Prime MM-GBSA energy are not calculated, as the poses are not accurate enough, but the docking score can be used to filter the poses. It can handle multiple chemical reactions within the same library, only requiring a generic SMARTS-based predefinition of the reaction. In a recent study, Liu et al. performed a customized SBVS using CovDock-VS to identify novel covalent CRM1 (chromosomal region maintenance 1) inhibitors. In the first step, a prefiltered database of ~30 000 Michael acceptors was docked to the target proteins using Glide Standard Precision (SP) mode. Subsequently, the top 1900 compounds were docked covalently using CovDock-VS. Among the 50 compounds that were tested based on visual inspection and application of different filters, AN-988 displayed a higher binding affinity (K_d_ = 615 nM) toward CRM1, as determined by the biolayer interferometry (BLI) assay [134].

#### DUckCov

DUckCov is a multistep VS protocol for the identification of novel covalent binders developed by Rachman et al, using constrained docking in combination with Dynamic Undocking (DUck) [135]. The protocol was prospectively validated in two case studies: a target with highly conserved noncovalent interactions (JAK3) and another one where the noncovalent interactions are not conserved across known inhibitors (KRas^G12C^). The authors first docked a ZINC acrylamide collection (~50 000) with rDock [136] against the target protein structures, while simultaneously tethering the covalent warhead to its reference coordinates and using pharmacophoric constraints to enforce the main noncovalent interaction. H-bond pharmacophoric constraints were set in the docking simulation to keep only those ligands that can establish the H-bond interactions defined as important for binding. Dynamic Undocking (DUck) [137] is then used to evaluate the strength of the H-bond. Lastly, only those ligands that display the best noncovalent interactions (according to rDock and DUck) are covalently docked with CovDock [128] using the most accurate pose prediction module. For JAK3, two novel covalent ligands were experimentally identified with a hit rate of 60%. For the KRas^G12C^ protein target, a low nanomolar inhibitor was identified with a hit rate of 44%.

#### Cov_FB3D

The Fragment-Based De novo Covalent Drug Design (Cov_FB3D) is a covalent inhibitor design protocol implemented by Wei et al [138]. In the first step, the warheads were covalently docked into the target protein by using AutoDock v4.2 [107, 108]. In the next step, noncovalent substructures were generated and docked into the active site of the target protein by using Surflex-Dock [139, 140], and the fragment poses with high docking scores were identified. Then, the noncovalent substructures were generated through the ‘in silico’ assembly of the best-predicted fragment poses. This was followed by performing Binding Affinity Score Across Multiple-level Potential energy (BA-SAMP) scoring. BA-SAMP is a combined scoring strategy, which integrates the score of the noncovalent part with X-Score [141] as the coarse level, and the score of the whole covalent compounds with the PM7 as the fine level to rank the correct constraint structure in the top 2% of top-level candidates. Finally, the synthetic accessibility of suggested covalent candidates was evaluated by machine-learning-based synthetic accessibility (SCScore) [142]. The Cov_FB3D protocol was successfully applied in the de novo design of an ALDOA inhibitor (T1) with potent inhibitory activity (0.34 ± 0.03 μM).

#### WIDOCK

WIDOCK is a VS protocol developed by Scarpino et al. [143] that supports the selection of diverse electrophiles as covalent inhibitors by incorporating ligand reactivity towards cysteine residues into AD4. It applies the reactive docking methodology described by Backus et al [144]. The docking approach does not involve the formation of a chemical bond between the ligand and the nucleophilic residue, but it focuses on the prediction of the noncovalent interactions occurring in the binding pocket before the covalent bond formation and uses a reactivity-scaled reward for compounds able to place the reactive group in the cysteine vicinity. The parameters of the potential were derived either from kinetic data measured in reactions of various small compounds against cysteine surrogates or from calculated quantum chemical reaction barriers. The performance of this docking protocol was first evaluated by investigating compound series with diverse warhead chemotypes against KRAS^G12C^, MurA, and cathepsin B. On KRAS^G12C^, WIDOCK was able to retrieve 10 out of the 12 experimental actives within the defined distance cutoff, that represents 83% sensitivity as compared to 75% achieved by AD4. While against MurA and CatB, WIDOCK achieved 60% and 100% sensitivity, respectively. Finally, WIDOCK was successfully applied in the identification of several covalent human MAO-A inhibitors acting by binding to Cys323.

### Covalent docking with attracting cavities

Goullieux et al. recently reported a new covalent docking procedure based on attracting cavities (AC), which mimics the two-step mechanism of covalent ligand binding [145]. The conformational sampling is first performed with the noncovalent prereactive topology of the ligand, followed by a pose-refinement and scoring with the postreactive topology, which includes the covalent bond with the protein and the appropriate stereochemistry of the complex. The authors termed this method a ‘switch’ method and compared its performance to results obtained with purely noncovalent docking (‘non-cov’) and with purely covalent docking, starting directly from the postreactive topology of the ligand (‘cov-only’). The covalent AC docking code, as well as the parameter and topology files, have been made available through the SwissDock Web server www.swissdock.ch [146, 147].

#### CovCIFDock

The CovCIFDock is a hybrid covalent pose prediction workflow based on the CHARMM-driven CIFDock flexible docking method [148, 149]. Protein-ligand complexes are docked classically in the prereactive state, after which a QM/MM minimization is conducted to form the protein-ligand bond and refine the final pose. CovCIFDock was validated against 46 protein receptors belonging to Cathepsin K, HCV NS3, EGFR, and XPO1 targets. Results showed that CovCIFDock was able to replicate the binding mode of these covalent complexes with RMSD <2 Å of the native conformation, and was comparable in accuracy to leading commercial docking programs.

### Artificial intelligence-guided covalent pose prediction tools

#### AlphaFold3

AlphaFold3 (AF3) is the latest AI-based all-atom prediction model of biomolecular structures from Google DeepMind [150]. AF3 facilitates high-accuracy prediction of noncovalent as well as covalent protein-ligand complexes (i.e. for a range of ligands but not all types of ligands for now, at least not with the open version) [151]. To probe the performance of AF3 in the covalent domain, the authors constructed a test dataset ‘COValid’ for the enrichment analysis of covalent VS. The capabilities of three physics-based classical covalent docking tools (DOCKovalent method [152] based on DOCK3.7, the attach-and-grow method based on DOCK6.12 [103], and the flexible sidechain method of AutoDock [107]) were also measured for a direct comparison with AF3. The prediction pipeline of AF3 was used to predict covalent complexes for all compounds in COValid using the protein sequence and geometrically optimized 3D conformer as input. The input also included the specification of the ligand atom and the residue atom to be covalently bonded. Finally, Rosetta [153, 154] was employed to minimize and energetically score all the predicted AF3 models, and used this score to calculate the enrichment performance across COValid. It is important to mention that presently, not all residues and warheads are supported in AF3. The study mainly focused on the most common electrophile acrylamide that targets cysteines. Based on the results achieved from AF3, the tool seems superior and recommended over classical covalent docking tools for the cysteine-acrylamide complex modeling.

#### Interformer

Interformer is a computational AI model designed to alleviate the interaction-aware problems in protein-ligand docking and employs constructive learning for affinity prediction [155]. Interformer is enabled with an interaction-aware mixture density network (MDN) that models noncovalent interactions, explicitly focusing on the hydrogen bonds and hydrophobic interactions present in the protein-ligand crystal structure. Also, a pseudo-Huber loss function is employed for leveraging the capabilities of contrastive learning to instruct the model in discriminating between favorable and unfavorable binding poses. The program is built on the Graph-Transformer framework, which has demonstrated its superior performance compared to GNN-based models in various graph representation learning tasks. Though Interformer specializes in modeling of noncovalent ligands, the program was successfully used in the Mpro covalent test set, where it achieved a correlation of 0.46, outperforming CovDock. In the Mpro project test set, Interformer significantly outperformed CovDock by a correlation of 0.604. The authors successfully used Interformer for optimizing covalent-type small molecules for the SARS-CoV-2 Mpro target. By using this tool, nine small molecules were developed, with the most potent one achieving an affinity of 16 nM.

#### CarsiDock-Cov

CarsiDock-Cov [156] is a DL-guided tool for covalent pose prediction based on the docking principles of CarsiDock [157]. The program comprises a DL model to forecast the protein–ligand atomic distance matrices and a geometric optimization stage to reshape the distances into a credible binding pose, for covalent docking. Similar to mainstream covalent docking programs, the program requires the specification of the nucleophilic residue in the protein, and the ligands to be docked are converted into the postreacted state. The program leverages the DL models trained for noncovalent complexes to handle covalent binders, thus ensuring model generalizability; incorporates covalent bond formation directly into the pose reconstruction stage to enforce geometric constraints; and integrates the core DL model with multiple auxiliary modules into a unified pipeline for streamlined automation. The docking poses are scored using CarsiScore, a built-in DL-based scoring function of CarsiDock, followed by the final binding strength estimation using RTMScore, a residue-atom distance likelihood potential. It is important to note that neither CasiScore nor RTMScore specifically takes the contributions of covalent attachment into account.

#### CovDocker

CovDocker is a benchmarking tool designed to advance DL driven covalent drug design [158]. The tool performs three main tasks: (i) Reactive location prediction, which involves identifying both the pocket in the protein and the specific residue site most likely to undergo a covalent chemical reaction; (ii) Covalent reaction prediction, which determines the product of a covalent reaction by predicting atom connectivity and functional groups formed between the residue and the molecule; and (iii) Covalent docking, which predicts the low-energy complex conformation of the covalently bound molecule within the extracted pocket. The covalent docking does not consider the energy contribution from the covalent bond formation. In the first step, Uni-Mol Block [159] is used for reactive location prediction, incorporating a cross-attention mechanism to accurately identify the potential pockets and target residue sites. For covalent reaction prediction, Chemformer [160] was fine-tuned as a sequence-based model to forecast covalent reaction outcomes. Finally, the Uni-Mol docking model was adapted to train a site-specific docking method with auxiliary covalent constraints to enhance binding accuracy.

### Benchmarking studies on covalent docking tools

To our knowledge, four research works have been reported that benchmarked the performance of a variety of available covalent-docking tools. However, it is important to mention that we are only considering comparative studies that include at least four different covalent docking tools. First was the study by Scarpino et al., where they evaluated six covalent-docking tools (FITTED, AD4, MOE, GOLD, COVDOCK, and ICM-Pro) against a dataset consisting of 207 protein-ligand complexes with diverse electrophilic warhead groups and receptor types [161]. At a 2 Å RMSD cut-off, MolSoft’s ICM-Pro software was able to achieve the experimental ligand pose in the top 1 conformation in 62% of the cases and the top 10 conformations in 88% of the cases. ICM-Pro software outperformed the other programs in all tests, including reproducing the experimental binding modes and scoring. ICM also performed well with more challenging flexible ligands with 35 heavy atoms or more. In an interesting side test described in the paper, it was found that ICM-Pro’s noncovalent conventional docking procedure also gives a remarkable rate of accurate predictions for covalent ligands to modified receptors (Cys/Ala mutations- 86% near native) [161]. Second was the study by Wen et al. assessed the performance of four covalent-docking programs (COVDOCK, MOE, ICM-Pro, and GOLD) by employing a dataset from the BCDE set, which consists of 330 diverse ligand scaffolds and 104 receptor targets. Based on the results, MOE was found to be better than other tools in terms of the best-scoring pose (with a median RMSD of 1.94 Å), and CovDock performed better than other tools in terms of the best sampled pose (with a median RMSD of 1.33 Å). Third was the study by Wei et al., in which they developed a hybrid covalent-docking method known as COV_DOX [162] (see Supplementary Information) and compared its performance to MOE, ICM-Pro, CovDock, and GOLD. Cov_DOX achieved an overall success rate of 81% with RMSD <2 Å for the top 1 pose prediction in the validation against a test set including 405 crystal structures of covalent protein-ligand complexes, covering various types of warhead chemistry and receptors. Although COV_DOX was validated to have a higher performance rate than all other covalent-docking tools, the hybrid method is considered ‘not feasible’ for a VS experiment because of its very high computing time, which is a result of its GSA (generalized simulated annealing) quantum mechanical calculation method. The fourth study was by Wu et al., in which they developed a covalent-docking webserver known as HCovDock (see Supplementary Information) and compared its performance to AutoDock, Cov_DOX, CovDock, FITTED, GOLD, ICM-Pro, and MOE. The program was tested on a dataset of 207 diverse covalent protein-ligand complexes. The results showed that HCovDock exhibits a significantly better performance than other covalent docking programs. With the criterion of ligand root-mean-squared distance <2.0 Å, HCovDock obtained a high success rate of 70.5% and 93.2% in reproducing experimentally observed structures for the top 1 and top 10 predictions. Although HCovDock has shown to have excellent performance and is relatively fast compared to other programs, the webserver cannot be used in a VS setting involving large covalent libraries. Based on the closer inspection of results reported in these studies, it seems that ICM-Pro and CovDock could be of interest in both covalent hits identification through VS and optimization stages, whereas Cov_DOX protocol fits more the optimization purpose. Tools GOLD and AD4 showed satisfactory performances across different datasets, indicating that both tools are still of interest. Particularly, AD4 in the situation where the user does not have access to the commercial tools. It is also important to mention that scoring functions are often target dependent, so the final choice for selecting a covalent docking tool should consider various aspects such as software availability, performance of scoring function in the protein of interest, chemical reaction to be performed, throughput of the method etc. Besides the significant performances achieved by some of the classical covalent docking tools, AF3 [151], an AI-guided covalent pose prediction tool, has been recently developed for performing covalent VS. AF3 achieved superior results as compared to conventional covalent docking tools against a large curated dataset of covalent protein-ligand complexes. Hence, AF3 seems very promising, and in the coming years, it will be interesting to observe how such AI-based tools will be applied by the community for the prospective identification of novel covalent binders. The results obtained in different comparative studies have been summarized in Table S2 (see supplementary information).

### Post-processing tools for docking analysis

Besides the mainstream covalent docking software, a growing number of DL-based tools for noncovalent docking have recently become available, and these tools could be used for the pose prediction of covalent binders in an unbound form that could be further postprocessed to generate covalent complexes. However, it will be important to assess the quality of the generated poses from these tools not only in terms of RMSD but also for chemical and geometric consistency, stereochemistry, and the physical plausibility of intra- and intermolecular measurements. To assist the process, Buttenschoen et al. developed a tool called PoseBusters, which is a Python package that performs a series of standard quality checks against the docking poses using the cheminformatics RDKit toolkit [163]. The authors used this tool to compare five DL-based docking methods (DeepDock [164], DiffDock [165], EquiBind [166], TankBind [167], and Uni-Mol [159]) and two conventional docking methods (AutoDock Vina and GOLD) with and without an additional postprediction energy minimization step using a MMs force field. The results indicated that, both in terms of physical plausibility and the ability to generalize to examples that are distinct from the training data, no DL-based method performed better than the classical docking tools. The authors noted that MM force fields contain docking-relevant physics that are missing within deep-learning methods, which might explain the decreased performance of these tools. A similar tool was developed by Harris et al. called PoseCheck [168] to assess the quality of generated molecules conditioned on a 3D protein pocket in terms of known physical constraints for binding and the extent to which redocking alters the generated interactions. After extensive analysis of PoseCheck on multiple state-of-the-art 3D generation methods, it was found that generated molecules have significantly more physical violations and fewer key interactions compared to baselines, calling into question the implicit assumption that providing rich 3D structure information improves molecule complementarity. Based on these studies it seems possible that the bad poses resulting from deep docking or 3D generative methods may be related to limited conformational sampling of the poses, and performing more rigorous exploration of the conformational landscape of the poses might be needed while this may not be required for classical tools (i.e. ~10–20 poses should be sufficient). However, this hypothesis requires further investigation, but it is important to keep in mind that one of the main objectives of these modern AI-based 3D prediction tools is to drastically reduce the computational simulations time compared to traditional methods using a backend model and any such limitations would make these tools less trustworthy unless they are further optimized. In fact, given the significant ongoing efforts in the field, the situation is expected to evolve in the coming months.

### Covalent docking webservers and structural databases

Besides the standalone commercial and free covalent docking programs, many user-friendly web-based tools are available to help scientists perform covalent docking experiments (see Supplementary Information). Examples include Cov_DOX [162, 169, 170], Kin-Cov [171], DOCKovalent [152], CovalentDock Cloud [172], HCovDock [173], SCARDock [174], CovPepDock [153, 175], HADDOCK 2.4 [176–179], and DockThor-VS [180–182]. The prominent databases of covalent ligands and 3D covalent protein-ligand complexes include Cysteinome [183], cBinderDB [184], CovalentInDB 2.0 [185], CovBinderInPDB [186], and CovPDB [187] (see Supplementary Information). These databases can be used for benchmarking covalent docking programs or cross-docking campaigns for repurposing existing covalent binders. The increasing availability of covalent docking webservers, curated inhibitor databases, and experimentally solved 3D structures of protein-covalent ligand complexes offers exciting opportunities to combine in silico calculations with experimental validation. Such integration holds promise for the discovery of novel covalent hits, some of which may evolve into viable drug candidates or serve as valuable chemical probes for exploring complex disease biology.

### Pharmacophore-based tools

In addition to docking, pharmacophore-based approaches can be used for the identification and rational design of covalent binders. The pharmacophore-based approaches can be grouped into three types: (i) the first type is ‘*holo*’ pharmacophore derived from an experimental 3D protein-ligand complex, which is based on probing the possible interaction points between the ligand and the target; (ii) the second type is pharmacophore derived from the bioactive ligands without consideration of the 3D structure of the target proteins for which many active molecules are superimposed to extract the common features that are crucial for activity; (iii) the third type is ‘*apo*’ pharmacophore generated from the predicted or known active site of a protein structure for which there is no known 3D ligand-bound structure. The *apo* pharmacophore can be used as a query to identify ligands that can be accommodated within this model with a good chemical features complementarity [52, 188, 189]. The major pharmacophore-based tools that can be used for modeling covalent ligands include LigandScout tools AncPhore [191], CSD-CrossMiner [192], Phase [193, 194], and Catalyst [195]. Some of these tools have implemented a covalent feature or allow customization to include a covalent feature in the query pharmacophore to account for electrophilic groups of the covalent ligands (see Supplementary Information for more details on these tools and the reported case studies).

## Discussion

While covalent binders hold significant promise in drug discovery, potential ADMET challenges should be carefully considered (see Supplementary Information). Despite these challenges, covalent binders remain highly relevant due to their ability to target proteins previously considered undruggable [196], offering therapeutic opportunities where reversible modulators often fail. In fact, covalent inhibitors belong to a broader set of emerging therapeutic strategies, including covalent biologics and targeted protein degradation technologies such as PROTACs [197] and molecular glues. Among covalent biologics, Antibody Drug Conjugates (ADCs) have gained attention [198], combining antibodies, cytotoxic drugs, and linkers to deliver chemotherapy selectively. Similar strategies apply to covalent nanobodies designed for irreversible binding [199], suggesting covalent modeling should play an increasing role in the coming years.

The identification of targeted covalent binders through large-scale experimental screening is difficult and costly. Covalent VS accelerates this by narrowing the search space. Yet it is important to note that many tools still have limitations, particularly in accurately evaluating covalent bond formation energies [2, 200]. This might impede the correct ranking of the compounds and subsequently impact the selection of molecules. Another important prerequisite before performing covalent VS is to prepare a prefiltered database based on chemical reactions, followed by standardization. Most tools can only be used to dock a small library due to the high CPU/GPU time needed for performing covalent computations and/or scoring (or rescoring with additional energy terms), rendering them unsuitable for performing VS. Also, the covalent docking tools cannot really predict the intrinsic reactivity of electrophiles, which may result in highly reactive compounds or false positive hits during VS. However, with more powerful computers, some tools can handle large libraries such as CovXplorer (SeeSAR), FLARE, FITTED, etc, while others integrate empirical models or QM calculations to account for covalent bond energetics (e.g. CovalentDock, HCovDock, WIDOCK, and Cov_DOX). Some programs have included a receptor desolvation term to improve the scoring, such as AD4, DOCK6, which uses a SASA-based approach to measure desolvation energy; BioSolveIT SeeSAR uses a hybrid desolvation model; and GOLD (ChemScore and PLP scoring functions), GlideScore uses empirical desolvation terms. MOE also calculates desolvation energy when poses are scored using the GBVI/WSA dG scoring function. CovDock includes MM-GBSA scoring that considers receptor desolvation in its pose prediction mode.

Another important aspect, particularly in the case of prochiral warheads, is the enumeration of different stereoisomers of the covalent complex. Some of the docking tools, such as MOE, ICM-Pro, and covalent docking with Attracting cavities, explicitly enumerate possible stereoisomers for the final scoring. In the situations where the tool is not taking into account stereochemistry, it is recommended to generate isomers using some subsidiary tools, such as ChemAxon (commercial) [201] or RDKit (open source toolkit) [202], in the postreacted state.

Some packages have incorporated a rigorous free energy calculation protocol to evaluate covalent ligands. For instance, the Schrodinger’s Free-energy perturbation (FEP^+^) module can successfully handle ligands that are covalently bonded to the receptor and show the remaining noncovalent interaction terms (hydrogen bonding, electrostatics, etc.) [203–205]. The FEP has not yet been implemented in conjunction with Schrodinger’s CovDock. In general, these types of free energy calculations cannot be used in VS settings and seem more appropriate for compound optimization. Promising approaches combine initial VS with physics-based affinity calculations, such as FEP [204, 206], or QM-based fragment molecular orbital (FMO) methods [207, 208]. On the other hand, protein flexibility remains a challenge. In situations where the receptor is known to be flexible or has flexible residues in the active site, ensemble docking can be achieved by docking a ligand to multiple conformations of the protein receptor [209, 210]. Optimizing side chains, rescoring poses, and employing MDs simulations could improve docking accuracy in some cases. AD4 covalent docking with flexible side chain method is one such tool that allows exploring protein conformations while performing covalent docking. In a recent study, Zhu et al. modified the CovDock program to allow for protein conformational mobility in the prediction of ligand binding pose and binding affinity. This new docking approach, termed FlexCovDock, improved success rates from 55 to 89% for binding pose prediction on a dataset of 10 cross-docking cases and successfully used in the structure-based design of KRAS^G12C^ inhibitors [211]. Tools like GOLD, allow limited on-the-fly flexibility, while Schrodinger’s IFD-MD (induced fit docking-molecular dynamics) module handles covalently bound ligands using combined docking, scoring, and MD-based sampling [212–217], though at high computational cost. Predicting intrinsic reactivity [218] can be valuable like for instance using Fukui indices. These are used to describe local chemical reactivity based on the frontier molecular orbitals (HOMO and LUMO) [219, 220]. One can rank the molecules according to these values or these values can be used in a consensus scoring approach [221, 222]. In addition, QSAR can also be used to predict electrophilicity based on QM descriptors.

An important prerequisite in the covalent drug discovery is the identification of a target residue in the active site with adequate accessibility and reactivity that can allow the close positioning of the ligand warhead near the nucleophilic side chain, followed by chemical bond formation. Some tools can predict the reactivity of cysteine residues, such as CovCys [223], SILCS-Covalent [224], ICM [87], Cpipe [225], BioLuminate [131, 226, 227], and HyperCys [228]. Analogous to the identification of reactive residues in the binding sites that can serve as potential nucleophiles, some recent studies attempt to identify electrophilic ligands from a large database of small molecules. For instance, Gil and Rowley et al. employed traditional and graph ML algorithms to classify molecules as reactive or nonreactive towards proteins [229] (see Supplementary Information). A potential tool based on these types of algorithms can be of great interest in covalent VS compared to traditional filtering methods that often use SMARTS patterns. Such tools could indeed speed up the search in ultra-large compound collections.

In parallel, new AI-driven methods such as co-folding models are emerging. Notably, AF3 [151] uses a diffusion-based model to predict the joint 3D structure of proteins and small molecules (co-folding). While the full AF3 model has the theoretical ability to generate the structure for any ligand, the public, free AlphaFold Server is restricted to a limited list of small molecules (like common cofactors, ions, etc), and for the time being, large-scale VS with novel or custom compounds is not allowed. Though not designed for covalent binding, AF3 has shown remarkable accuracy, achieving near-perfect classification for covalent active compounds, but this success has primarily been demonstrated on well-studied targets like kinases [151], and its generalization to protein families with sparse covalent binder data is still an area of active research. Besides AF3, other generative frameworks like Boltz-2 [230] show potential for modeling diverse ligand types. Also, AI-based 3D generative programs (ligand and pocket-based), such as LibINVENT [231] and Diffleop [232], can be used to generate novel covalent molecules compatible with downstream covalent docking. Integration of such AI tools into covalent drug discovery could expand opportunities.

**Figure 4:**
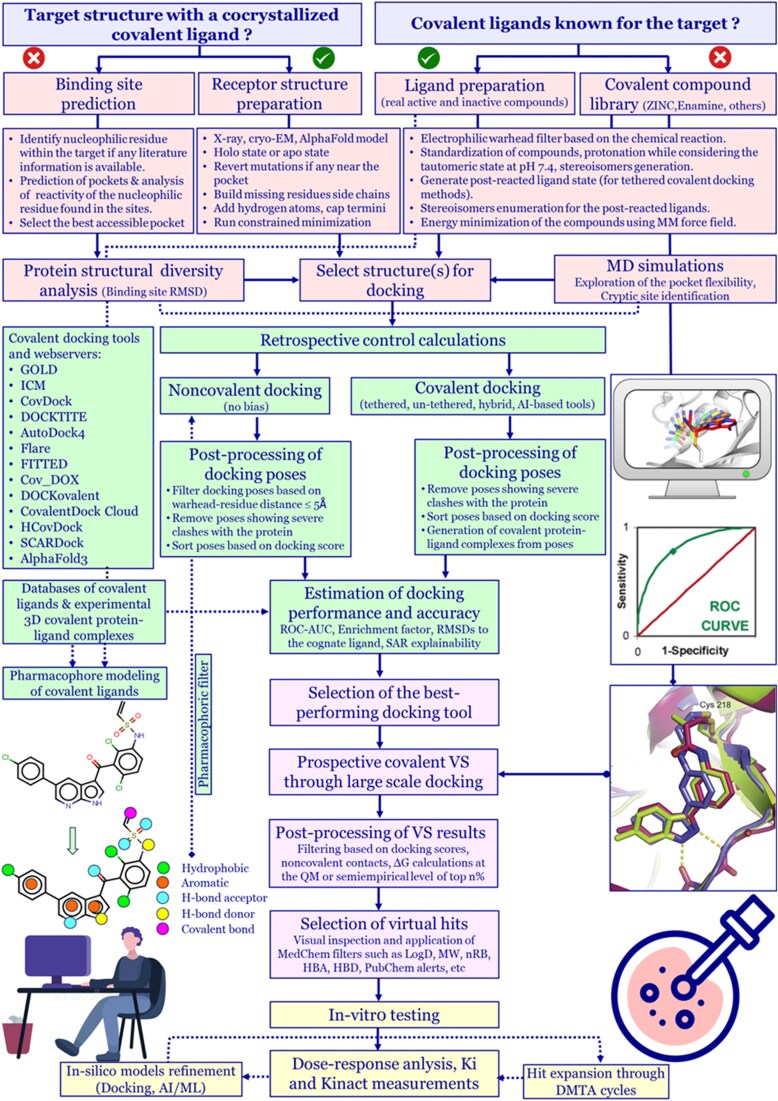
An example of a structure-based workflow for benchmarking different covalent docking tools and the discovery of novel covalent hits through prospective VS. Various stages of the workflow are highlighted in different colors: light blue regions corresponds to the availability of 3D protein structures, target residue information, and known covalent binders of the target; light red regions corresponds to selection of the target protein structure, protein preparation and ligand preparation; light green regions corresponds to retrospective assessment of docking success and accuracy; light purple regions corresponds to prospective covalent VS; and light yellow regions corresponds to experimental testing and hit expansion through DMTA cycles.

This review summarizes available docking programs, pharmacophore tools, covalent ligand databases, and cysteine-reactivity predictors, guiding researchers in selecting appropriate tools. These approaches enable identification of novel hits and can also be adapted for drug repositioning [233], target-fishing through reactive docking [70], or for studying drugs’ adverse effects, and prediction of drug–drug interaction. We provide a workflow template to combine different strategies depending on project needs (Fig. 4). For instance, considering a situation where the 3D structure of a target with a cocrystallized covalent inhibitor is available along with some known binders, users could benchmark the ability of different covalent docking tools [161, 234] or use pharmacophore-based approaches to assess the accuracy in reproducing the bioactive conformation as well as the ability of the different tools to discriminate between actives and inactives [235]. If users have access to only one noncovalent docking tool, then the focus should be on retrieving poses showing optimal distance between the warhead and reactive residue, and compatible noncovalent interactions compared to a cognate binder. In fact, David et al. showed that noncovalent docking of covalent ligands by GOLD performed similarly to covalent docking in terms of AUC, though with reduced enrichment factors [236], indicating that noncovalent docking tools could also be used to simulate covalent compounds. In another study, Basse et al. used Surflex [139, 140] to dock Bortezomib in the proteasome to compare the orientation of noncovalent molecules that were screened by docking and then tested experimentally [237]. The authors were able to retrieve a good orientation of Bortezomib in the pocket, suggesting that in some cases, a regular docking engine and normal scoring function can be used to investigate covalent binders. When experimental structures are unavailable, predicted 3D models (e.g. AlphaFold [150]) can be used, though with caution. Emerging workflows now integrate structure generators, multiparametric optimization, in silico pharmacological and ADMET models, PB/PK predictions, and systems pharmacology [238], offering a comprehensive view of disease mechanisms and drug effects.

Besides the conventional covalent docking workflow, efforts have been made towards developing tools that can help transform a reversible binder into a covalent binder. For instance, Zaidman et al. developed a computational pipeline termed covalentizer for identifying irreversible inhibitors based on structures of targets with noncovalent binders. By using this tool and covalent docking-based screening, the authors discovered five novel covalent kinase inhibitors with IC_50_ values between 155 nM and 4.5 μM. The tool was applied against an existing SARS-CoV M^pro^ reversible inhibitor that led to an acrylamide inhibitor with low μM affinity against SARS-CoV-2 M^pro^ [239]. The program is accessible at https://github.com/LondonLab/Covalentizer. Another recent example includes CovalentLab [240], which is an integrated platform for the rational design of covalent molecules from noncovalent ligands. The program identifies nucleophilic residues in target binding sites, optimizes warhead attachment points, and integrates warheads into ligands. It also supports custom warhead design by matching suitable ligands and targets for specific user-defined warheads. CovalentLab supports nine amino acid targets (Asp, Arg, His, Cys, Ser, Lys, Glu, Thr, and Tyr) and features a curated library of 220 diverse warheads. In the platform’s library section, several targets have been investigated to illustrate the potential of the method and this collection covers 95 key targets, 302 reversible protein-ligand complexes, and 109 903 covalent inhibitors. The platform and database section are fully accessible to the public at https://www.medchemwise.com/CovalentLab.

As seen above, when describing the different docking engines, covalent or reversible covalent binders have demonstrated significant potential in addressing protein targets traditionally considered undruggable or difficult by conventional, fully reversible small-molecule drugs (e.g. targets such as the proteasome, mutant KRAS^G12C^, BTK). Several covalent or reversible covalent drugs are indeed FDA approved, for instance, Ibrutinib (BTK), Osimertinib (mutant EGFR), Bortezomib (proteasome), and Carfilzomib (proteasome). A notable other example of this strategy is the identification of a covalent ligand, EN4, that targets a cysteine residue within the intrinsically disordered region of the oncogenic transcription factor c-Myc [241]. Covalent bifunctional molecules are also rapidly gaining momentum as a strategy for developing highly specific and potent agents capable of modulating protein function with unprecedented precision [242].

In conclusion, we hope this review can serve as a practical resource for medicinal chemists and experimental scientists, highlighting computational tools that should accelerate the discovery of covalent binders. The growing availability of docking servers, curated databases, and structural data enables synergy between in silico predictions and experimental validation. Looking ahead, integration of big data, AI, and physics-based methods is expected to drive innovation in covalent drug discovery further [243–255].

**Key Points**

- We report conventional and modern AI-driven covalent docking tools; and covalent docking webservers. Besides docking-based tools, we also report pharmacophore-based tools, and small molecule and 3D structural databases related to covalent ligands.
- Based on the description of the tools, experimentalists should be able to identify the docking tools and services that best suit their needs.
- Given the limitation associated with most of the docking tools due to ignorance of covalent bond energy contribution, it is recommended to perform *post hoc* QM/semi-empirical calculations, rescoring with different scoring functions, or consensus scoring of the resulting poses.
- This survey could also be valuable to research groups and scientists engaged in the identification and optimization of covalent binders.

## Supplementary Material

supplementary_information_covalent_docking_tools_review_revised_NS_et_al_Nov-13-2025_bbaf697

## Data Availability

This review is based solely on previously published studies. No new datasets were generated. All extracted information is provided in the manuscript and supplementary tables.
