# Supplementary material for: The covalent docking software landscape: features and applications in drug design
Source: Brief Bioinform. 2025 Dec 31;26(6):bbaf697. doi: 10.1093/bib/bbaf697 (PMC12753312; doi:10.1093/bib/bbaf697)
Supplement: supplementary_information_covalent_docking_tools_review_revised_NS_et_al_Nov-13-2025_bbaf697 [file supplementary_information_covalent_docking_tools_review_revised_ns_et_al_nov-13-2025_bbaf697.docx]

**Supplementary Information**

**The Covalent Docking Software Landscape: Features and Applications in Drug Design**

Natesh Singh^1, *^, Philippe Vayer^2^, Bruno O. Villoutreix^2,*^

*^1^Evotec SE, Molecular Architects, Integrated Drug Discovery, Campus Curie, Toulouse, France*

*^2^Université Paris Cité, Inserm UMR 1141, Hopital Robert-Debré, Paris, France*

Corresponding Authors:

Natesh Singh [natesh.singh@evotec.com](mailto:natesh.singh@evotec.com)

Bruno O. Villoutreix [bruno.villoutreix@inserm.fr](mailto:bruno.villoutreix@inserm.fr)

**Binding Kinetics of Covalent Ligands**

The first step of the binding event of a covalent ligand is a reversible process, while the second step can be reversible or irreversible depending on the free energy profile of the covalent reaction. **Figure S1** illustrates this two-step mechanism of covalent ligands that has been adapted from the references [1–3]. First, the ligand (I) binds to the target protein (P), and a reversible protein-ligand complex [P⋅⋅⋅L] is formed. The potency of this first step is defined by the binding constant K_I_. The K_I_ term describes the concentration of inhibitor required for half of the maximum potential rate of covalent bond formation. This is different from K_i_ describing the dissociation of the P⋅⋅⋅I complex, which is not affected by covalent bond formation. In the second step, the nucleophile reacts with the electrophile, forming a covalent protein-ligand complex [P−L]. The rate of the second step depends on the concentration of [P⋅⋅⋅L]. When all the target protein exists with reversibly bound ligand [P⋅⋅⋅L] and no free target protein remains (P), the observed rate of inactivation is the k_inact_. Taken together, the overall rate of covalent bond formation from free, unbound protein (P) to the covalent protein-ligand complex [P−L] is defined by the ratio of k_inact_ to K_I_ [2] (k_inact_/K_I_ , M^-1^ s^-1^ ) [4]. Thus, k_inact_/K_I_ ratio defines the kinetics of covalent bond formation and is considered a key component of covalent drug discovery through identifying, optimizing, and advancing the best and most selective ligands. Both non-covalent and covalent bonding events are associated with a free energy difference (ΔG^◦^_NC_ and ΔG^◦^_C_), governing the thermodynamics of the binding process. ΔG^◦^_NC_ is the free energy of binding resulting from the noncovalent interaction of the ligand with the protein, and ΔG^◦^_CB_ is the free energy difference resulting from the covalent binding of the ligand. The non-covalent and covalent binding processes are associated with the free energy of activation (ΔG^‡^_NC-f_ and ΔG^‡^_C-f_, for the forward reaction; ΔG^‡^_NC-r_ and ΔG^‡^_C-r_, for the reverse reaction, where ΔG^‡^_NC-r_ = ΔG^‡^_NC-f_  + ΔG^◦^_NC_ and ΔG^‡^_C-r_ = ΔG^‡^_C-f_  + ΔG^◦^_C_), determining the kinetic profile of the covalent ligand. The residence time of the covalent ligand upon non-covalent and covalent binding is related to them by ΔG^‡^_NC-r_ and ΔG^‡^_C-r_, respectively. The covalent reaction proceeds with a reaction free energy ΔG^◦^_C_ and requires the activation free energy ΔG^‡^_C-f_ to occur. ΔG^‡^_C-f_ determines the “reactivity” of the warhead: the lower the barrier ΔG^‡^_C-f_, the faster the reaction and vice versa. The magnitude of ΔG^‡^_C-r_ (i.e., the barrier for the reverse reaction) determines the reversibility of the covalent reaction. The ΔG^‡^_C-r_  is small in the cases of reversible covalent ligands, whereas it is essentially large in irreversible covalent compounds. An ideal modeling tool for covalent ligands should allow estimation of these four parameters (ΔG^◦^_NB_, ΔG^◦^_CB_, ΔG^‡^_NC-f_, and ΔG^‡^_C-f_). This would enable a more thorough comparison with the experimental inhibition data and could drive the rational design of covalent inhibitors[1]. Based on these kinetic determinants, the fine-tuning of covalent ligands would be possible. For example, by making ΔG^◦^_NC_ more exothermic and thermodynamically favorable by improving the noncovalent interactions and/or by increasing the residence time of the ligand in the noncovalent association complex (i.e., by raising the barrier of reverse reaction ΔG^‡^_NC-r_  through destabilization of the transition state), which in turn would increase the chance for the covalent reaction to occur even with weakly reactive systems (i.e., with relatively high ΔG^‡^_C-f_). This could improve the selectivity of the covalent ligand. Furthermore, the reversibility may be tuned by the interplay of ΔG^‡^_C-f_ and ΔG^◦^_C-r_. For instance, Bradshaw et al, by using an inverted orientation of the cysteine-reactive cyanoacrylamide electrophile identified potent and selective BTK inhibitors that demonstrated biochemical residence times spanning from minutes to 7 days [5]. Therefore, unlike in the rational design of conventional reversible ligands, covalent protein modulation is largely governed by reaction kinetics rather than binding thermodynamics, and concepts such as how long the bond forms, the percentage occupancy in the active site, and specificity or off-target binding are some of the important factors to be considered in the design of covalent molecules.


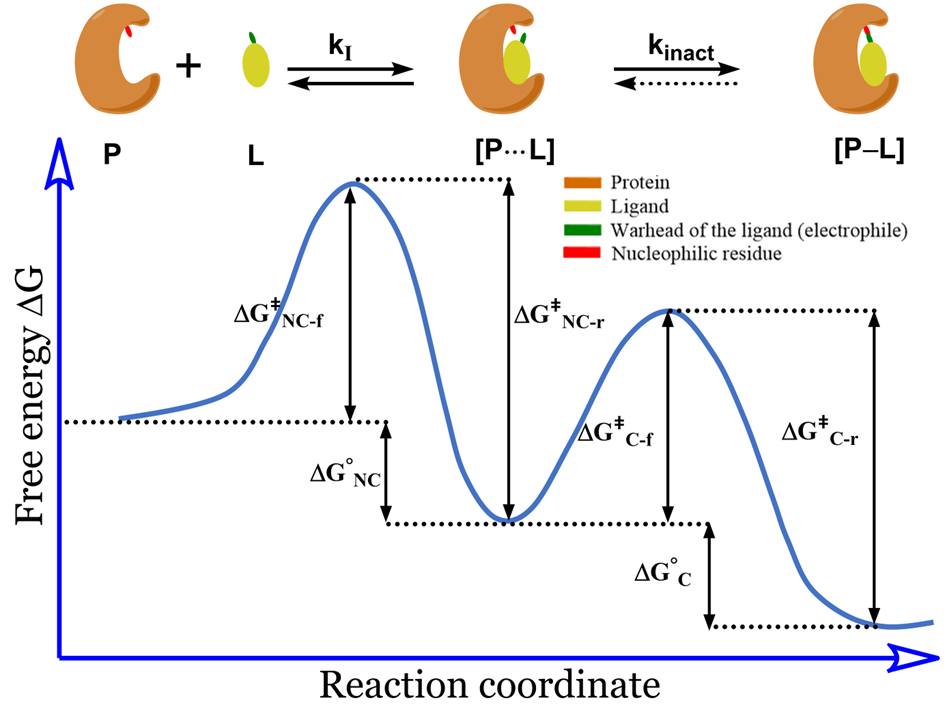


**Figure S1.** Model for two-stage covalent modification of a target protein (P) by a covalent ligand (L). [P⋅⋅⋅L] represents the initial non-covalent complex formed between P and L; [P–L] represents the covalent adduct.

| **Covalent docking tools** | | | | | | | | |
| --- | --- | --- | --- | --- | --- | --- | --- | --- |
| **Software** | **Method** | **Software format** | **Availability** | **URL** | **Covalent bond energetics** | **Residues supported** | **Speed/Throughput** | **Reference** |
| GOLD | Tethered | Standalone | Commercial | <https://www.ccdc.cam.ac.uk/solutions/software/gold/> | No | Ser, Thr,  Tyr, Lys, Cys, His, Arg; reactions can be added manually to target the residue of interest | Speed: customizable from fast to more accurate.  Throughput: high when jobs are run in parallel on an HPC | [6–8] |
| DOCKTITE | Tethered | Standalone | Commercial | https://www.chemcomp.com/Products.htm | No | Cys, Ser, Thr; reactions can be added manually to target the residue of interest | Speed customizable (the relative amount of time allowed for the placement of each ligand);  Throughput: high when jobs are run in parallel on an HPC | [9] |
| ICM-pro | Tethered | Standalone | Commercial | https://www.molsoft.com/products.html | No | Cys, Ser; reactions can be added manually | An average docking time is 0.5 seconds to 30 seconds per ligand per processor (accuracy can be increased using Docking_thoroughness parameter)  Throughput: high when jobs are run in parallel on an HPC | [10] |
| FlexX | Tethered | Standalone | Commercial | https://www.biosolveit.de/products/#FlexX | No | Most covalent warheads and  targeted residues are supported (ex. Cys, Ser) | Speed customizable (from more aaccurate binding mode prediction to docking of gigantic libraries through ultra-high-speed docking (< 1 s/ligand)  Throughput: a library of ~1,000 compounds can be screened in less than an hour on a laptop with 8 cores | [11–13] |
| Flare | Tethered | Standalone | Commercial | <https://cresset-group.com/software/flare/> | No | Cys, Lys, Tyr, Ser, Thr | Speed: customizable (Various docking modes: Normal, VS, Extra precision)  Throughput: high when jobs are run in parallel on an HPC | [14] |
| MacDOCK | Tethered | Standalone | Free | <https://dock.compbio.ucsf.edu/> | No | Ser | No information found on speed or throughput | [15,16] |
| CovalentDock | Tethered | Standalone | Free | <https://github.com/whbpt/covalentdock> | Yes | Cys, Ser | No information found on speed or throughput | [17] |
| AutoDock4 (flexible side chain method) | Tethered | Standalone | Free | <https://autodock.scripps.edu/download-autodock4/> | No | Cys, Ser, Thr, Lys | Speed: fast | [18,19] |
| GNINA 1.3 | Tethered | Standalone | Free | <https://github.com/gnina/gnina> | No | All covalent warheads and  targeted residues are supported | Speed: customizable from default CNN to fast; GPU dependent | [20] |
| DOCK 6 | Tethered | Standalone | Free | <https://dock.compbio.ucsf.edu/DOCK_6/index.htm> | No | Cys | Speed: fast with MPI-parallelization | [21] |
| AutoDock4 (two-point attractor method) | Untethered | Standalone | Free | <https://autodock.scripps.edu/download-autodock4/> | No | Cys, Ser, Thr, Lys | Speed: fast | [18,19] |
| CDOCKER | Untethered | Standalone | Commercial | [https://github.com/wyujin/Covalent-Docking-in-CDOCKER http://charmm.chemistry.harvard.edu/](https://github.com/wyujin/Covalent-Docking-in-CDOCKER) | Yes | Cys | average runtime for docking one covalent compound is 15 minutes | [22] |
| OpenDock | Untethered | Standalone | Free | <https://github.com/guyuehuo/opendock> | No | Not applicable | Speed: fast | [23] |
| Cov_FB3D | Hybrid | Standalone | Free | <http://202.114.32.71:10280/wandox/Cov_FB3D/home.html> | No | Cys, Ser | Speed: slow based on the docking protocol | [24] |
| DUckCov | Hybrid | Standalone | Free | <https://github.com/CBDD/rDock>  <https://github.com/CBDD/Duck> | No | Cys | Speed: slow based on the docking protocol | [25] |
| CovXplorer (SeeSAR 11.2) | Hybrid | Standalone | Commercial | <https://www.biosolveit.de/2021/11/24/covalent-docking-software-update-seesar-11-2-and-covxplorer-workflow/> | No | Most covalent warheads and  targeted residues are supported (ex. Cys, Ser) | Speed: fast | [11–13] |
| CovDock | Hybrid | Standalone | Commercial | https://www.schrodinger.com/life-science/learn/white-papers/covdock/ https://www.schrodinger.com/platform/products/glide/ | No | All covalent warheads and  targeted residues are supported | 1~2 hour per ligand | [26] |
| CovDock-VS | Hybrid | Standalone | Commercial | https://www.schrodinger.com/life-science/learn/white-papers/covdock/ https://www.schrodinger.com/platform/products/glide/ | No | All covalent warheads and  targeted residues are supported | ~ 15 minutes per ligand  Throughput: high when jobs are run in parallel on an HPC | [27] |
| Attracting Cavities | Hybrid | Standalone | Free | [www.swissdock.ch](http://www.swissdock.ch/) | No | Cys, Ser, Lys, His, Tyr, Glu, Asp, Met | Speed: Slow  Throughput: high when jobs are run in parallel on an HPC | [28] |
| WIDOCK | Hybrid | Standalone | Free | <https://autodock.scripps.edu/download-autodock4/> | Yes | Cys | Speed: slow based on the docking protocol | [29] |
| FITTED | Hybrid | Standalone | Commercial | <https://www.samson-connect.net/extensions/b72505d0-6942-ac90-2a78-b81b9d0cc5a1> | No | Cys | Speed: fast | [30–32] |
| CovCIFDock | Hybrid | Standalone | Free | URL not reported | No | Cys, Ser | Speed: slow based on the docking protocol | [33] |
| AlphaFold3 | AI-guided | Standalone | Free | <https://github.com/google-deepmind/alphafold3> | No | Cys | Average run-time per ligand (Sec.) for AF3 modeling: 266 ± 114 | [34] |
| CarsiDock-Cov | AI-guided | Standalone | Free | <https://github.com/sc8668/CarsiDock-Cov> | No | Cys, Ser, Lys | Speed: Fast | [35] |
| CovDocker | AI-guided | Standalone | Free | <https://github.com/PoloWitty/CovDocker/> | No | Cys, Ser, Lys, Thr, Glu, Asp, His, Tyr, Arg, Met | Speed: Fast | [36] |
| Interformer | AI-guided | Standalone | Free | <https://github.com/tencent-ailab/Interformer> | Not applicable | Not applicable | Speed: Fast | [37] |
| Cov_DOX | Hybrid | Webserver | Free | <http://doxwebserver.ccnu.edu.cn/covalent/index> | Yes | Cys, Ser | Speed: slow based on the docking protocol | [38] |
| Kin-Cov | Hybrid | Webserver | Free | <https://cki-builder.kincov.cn>  <https://github.com/Zhou-Yang/kincov> | No | Cys | Speed: Fast | [39] |
| DOCKovalent | Tethered | Webserver | Free | <http://covalent.docking.org/> | No | Cys, Ser | Speed: Fast | [40] |
| CovalentDock Cloud | Tethered | Webserver | Free | <http://docking.sce.ntu.edu.sg/> | Yes | Cys, Ser | No information found on speed or throughput | [41] |
| HCovDock | Tethered | Webserver | Free | <http://huanglab.phys.hust.edu.cn/hcovdock/> | Yes | Cys | Speed: 5 min average docking time; 1 sec for ligands with one rotatable bond; and 18 min for ligands with 23 rotational bonds | [42] |
| SCARdock | Untethered | Webserver | Free | [https://scardock.com/#/ http://www.liugroup.site/scardock/](https://scardock.com/#/) | No | All covalent warheads and  targeted residues are supported | Speed: Fast | [43] |
| CovPepDock | Untethered | Webserver | Free | <https://rosie.rosettacommons.org/cov_pep_dock/submit> | No | Cys | Speed: Slow | [44,45] |
| HADDOCK2.4 | Hybrid | Webserver | Free | <https://rascar.science.uu.nl/haddock2.4/> | No | Cys | Speed: slow based on the docking protocol | [46–49] |
| DockThor-VS | noncovalent docking | Webserver | Free | <https://dockthor.lncc.br/v2/> | No | Not applicable | Speed: Fast | [50–53] |
| **Pharmacophore-based tools for modeling covalent binders** | | | | | | | | |
| **Software** | **Method** | **Software format** | **Availability** | **URL** | | | | **Reference** |
| LigandScout | Pharmacophore-based | Standalone | Commercial | <https://www.inteligand.com/ligandscout/> | | | | [54–56] |
| AncPhore | Pharmacophore-based | Standalone | Free | <https://ancphore.ddtmlab.org/> | | | | [57] |
| CSD-CrossMiner | Pharmacophore-based | Standalone | Commercial | <https://www.ccdc.cam.ac.uk/solutions/software/csd-crossminer/> | | | | [58] |
| Phase | Pharmacophore-based | Standalone | Commercial | https://www.schrodinger.com/platform/products/phase/ | | | | [59,60] |
| Catalyst | Pharmacophore-based | Standalone | Commercial | https://www.3ds.com/products/biovia/discovery-studio/ligand-and-pharmacophore-based-design | | | | [61] |
| **Databases of covalent ligands and 3D covalent protein-ligand complexes** | | | | | | | | |
| **Database name** | **Number of proteins** | | **Number of covalent binders** | **Availability** | **URL** |  | **Reference** | |
| Cysteinome | 462 proteins with targetable cysteine residues from 122 different species | | 1217 | Free | <http://www.cysteinome.net/Cysteinome/index.php/Cysteinome> | | [62] | |
| CovalentInDB 2.0 | 368 | | 3445 | Free | <http://cadd.zju.edu.cn/cidb/> | | [63] | |
| CovBinderInPDB | 1170 | | 2189 | Free | <https://yzhang.hpc.nyu.edu/CovBinderInPDB> | | [64] | |
| CovPDB | 733 | | 1501 | Free | <http://www.pharmbioinf.uni-freiburg.de/covpdb/> | | [65] | |

**Table S1**: Computational and web-based tools based on covalent docking and pharmacophore modeling.

| **Comparative study 1** | | | | | |
| --- | --- | --- | --- | --- | --- |
| No. | Receptor types  (Scarpino et al.) [66] | Number of tested covalent complexes | Top 1 pose  (average RMSD) | Top 10 pose  (average RMSD) | Reference |
| 1 | Hydrolase | 126 | ICM-Pro (2.65 Å) | ICM-Pro (1.32 Å) | [66] |
| 2 | Transferase | 53 | ICM-Pro (1.28 Å) | ICM-Pro (1.03 Å) | [66] |
| 3 | Ligase | 4 | FITTED (1.46 Å) | FITTED (0.96 Å) | [66] |
| 4 | Metal binding protein | 3 | AutoDock4 (1.91 Å) | CovDock (1.15 Å) | [66] |
| 5 | Oxidoreductase | 3 | AutoDock4 (0.87 Å) | AutoDock4 (0.73 Å) | [66] |
| 6 | Transcription | 4 | MOE (1.39 Å) | FITTED (0.58 Å) | [66] |
| **Comparative study 2** | | | | | |
| No. | Receptor types  (Wen et al.) [67] | Number of tested  covalent complexes | Software with best scored pose  (average RMSD) | Software with best sampled pose  (average RMSD) | Reference |
| 1 | Hydrolase | 204 | CovDock (1.71 Å) | CovDock (1.39 Å) | [67] |
| 2 | Transferase | 83 | CovDock (1.3 Å) | CovDock (1.06 Å) | [67] |
| 3 | Ligase | 3 | CovDock (1.08 Å) | CovDock (0.84 Å) | [67] |
| 4 | Lyase | 1 | MOE (0.96 Å) | MOE (0.9 Å) | [67] |
| 5 | Oxidoreductase | 6 | ICM-Pro (1.13 Å) | GOLD (1.06 Å) | [67] |
| 6 | Isomerase | 6 | CovDock (1.02 Å) | GOLD (0.78 Å) | [67] |
| 7 | Transcription | 18 | CovDock (2.15 Å) | MOE (1.6 Å) | [67] |
| 8 | Viral protein | 4 | GOLD (0.81 Å) | GOLD (0.81 Å) | [67] |
| 9 | Metal binding protein | 5 | MOE (1.72 Å) | MOE (1.04 Å) | [67] |
| **Comparative study 3** | | | | | |
| No. | Number of tested  covalent complexes (Wu et al.) [42] | Docking program | Success rate when top 1  pose considered | Success rate when top 10 poses considered | Reference |
| 1 | 207 covalent  protein-ligand complexes | HCovDock | 40.6% (RMSD < 1 Å) 70.5% (RMSD < 2 Å) | 61.8% (RMSD < 1 Å) 93.2% (RMSD < 2 Å) | [42] |
|  |  | Cov_DOX | 19.8% (RMSD < 1 Å) 61.4% (RMSD < 2 Å) | 37.7% (RMSD < 1 Å) 92.8% (RMSD < 2 Å) | [42] |
|  |  | ICM-Pro | 32.9% (RMSD < 1 Å) 61.8% (RMSD < 2 Å) | 42.0% (RMSD < 1 Å) 88.0% (RMSD < 2 Å) | [42] |
|  |  | CovDock | 32.4% (RMSD < 1 Å) 58.9% (RMSD < 2 Å) | 49.8% (RMSD < 1 Å) 74.4% (RMSD < 2 Å) | [42] |
|  |  | AutoDock | 16.4% (RMSD < 1 Å) 55.1% (RMSD < 2 Å) | 29.5% (RMSD < 1 Å) 75.4% (RMSD < 2 Å) | [42] |
|  |  | GOLD | 23.7% (RMSD < 1 Å) 53.1% (RMSD < 2 Å) | 37.2% (RMSD < 1 Å) 65.2% (RMSD < 2 Å) | [42] |
|  |  | FITTED | 20.3% (RMSD < 1 Å) 47.8% (RMSD < 2 Å) | 39.6% (RMSD < 1 Å) 69.6% (RMSD < 2 Å) | [42] |
|  |  | MOE | 14.5% (RMSD < 1 Å) 37.2% (RMSD < 2 Å) | 22.7% (RMSD < 1 Å) 50.7% (RMSD < 2 Å) | [42] |
| **Comparative study 4** | | | | | |
| No. | Number of tested  covalent complexes (Wei et al.) [38] | Docking program | Success rate when top 1  pose considered (RMSD < 2 Å) | Success rate when best pose considered (RMSD < 2 Å) | Reference |
| 1 | 405 covalent  protein-ligand complexes (206 complexes used in Scarpino et al's work + 199 complexes from wen et al's work) | MOE | 46% | 67% | [38] |
|  |  | GOLD | 46% | 62% | [38] |
|  |  | CovDock | 58% | 72% | [38] |
|  |  | ICM-Pro | 53% | 75% | [38] |
|  |  | Cov_DOX(PM7 Top1) | ~ 58% | - | [38] |
|  |  | Cov_DOX(XO Top1) | 81% | - | [38] |
|  |  | AutoDock4 | - | 58% | [38] |
|  |  | Cov_DOX(GSA Top300) | - | 86% | [38] |
|  |  | Cov_DOX(PM7 Top10) | - | ~ 90% | [38] |

**Table S2:** Different comparative studies reported in literature on benchmarking of covalent docking tools.

**Web servers for performing covalent docking**

**Cov_DOX**

Cov_DOX is a hybrid method for performing covalent docking developed by Wei et al [38]. This tool is built on the DOX protocol that was originally designed for structure prediction of noncovalent protein-ligand bindings [68,69]. The Cov_DOX tool can handle structure prediction for covalent protein-ligand complexes. It incorporates a new generalized simulated annealing (GSA) technique as a sampling method at the coarse level [70]. The previously used medium-level theory (semi-empirical PM7) [71], and fine-level theory (DFT-based eXtended ONIOM (XO) [72] have also been reformed to adapt to the covalent ligands. Cao et al used COV_DOX for designing covalent inhibitors that target the allosteric AMP cavity of Fructose-1,6-bisphosphatase (FBPase) through noncovalent interactions as well as covalently bind to residue Cys179 to improve the target selectivity or long-lasting efficacy of AMP-site-directed inhibitors Structure-based optimization led to the discovery of a potent hit among other with an FBPase IC50 of 0.53 μM, Ki of 1.78 (μM), Kinact of 0.0012 (S^-1^) and Ki/Kinact of .67 (M^-1^.S^-1^) [73]. The Cov_DOX program can be accessed through a web server <http://doxwebserver.ccnu.edu.cn/covalent/index>.

**Kin-Cov**

Kin-Cov is a web server that has an integrated computational workflow (Kin-Cov) for the rational design of Covalent kinase inhibitors (CKIs) [39]. In Kin-Cov, a structure of a reversible inhibitor complexed with a kinase protein is used as the input. The complex structure can be obtained from the Protein Data Bank (PDB) or MD simulations. Based on the p*K*_a_ values obtained from the constant pH molecular dynamics (CpHMD) simulations, the most reactive cysteine at around 10Å of the reversible inhibitor is selected. The distances between the heavy atoms on the Murcko scaffold of the inhibitor and the thiol sulfur on the selected cysteine are measured to provide *d*_site_. Additionally, the *d*_site_ values can be obtained from the REST2 simulations. Next, different lengths of electrophilic warhead fragments (EWFs) in the library were added to the Murcko scaffold of the reversible inhibitor according to *d*_site_, and the generated candidates with unfavorable EWFs (*d*_esti_ > 5Å or *d*_esti_ < −5Å) were filtered out. In the web server, Autodock was applied for covalent docking based on the methods proposed by Bianco et al [18,74]. The receptor maps were calculated with AutoGrid, with the binding site for docking defined as a grid that includes the ligand and the reactive site. By using this workflow authors designed the first covalent leucine-zipper and sterile-α motif kinase (ZAK) inhibitor, showcasing the power of computational workflow for CKI design. The best two compounds inhibited ZAK kinase with half-maximal inhibitory concentration (IC_50_) values of 9.1 and 11.5 nM, respectively. The first displayed an excellent ZAK target specificity in Kinome profiling against 378 wild-type kinases. The Kin-Cov workflow and the developed tool are freely available at [https://cki-builder.kincov.cn](https://cki-builder.kincov.cn/) and<https://github.com/Zhou-Yang/kincov>.

**DOCKovalent**

DOCKovalent is a covalent VS web server, and it allows for the screening of large virtual libraries of electrophilic small molecules [40] by providing a structural model of a protein and a target nucleophile. This is an adaptation of DOCK 3.6 [75], allowing for covalent docking and targeting protein’s nucleophiles such as cysteine residues. Nine libraries of ligands bearing different electrophiles, amounting to over 650,000 commercially available or synthetically accessible small molecules, were developed for use with the method. Given a pre-generated set of ligand conformations and a covalent attachment point in the target protein, it exhaustively samples ligand conformations around the covalent bond and selects the lowest energy pose using a physics-based energy function. It was applied to discover reversible covalent fragments that target distinct protein nucleophiles, including the catalytic serine of AmpC β-lactamase and noncatalytic cysteines in RSK2, MSK1, and JAK3 kinases. In all cases, the hit-rates were high (5/6, 5/8, and 9/15 respectively) and the best inhibitors had better than 50nM potencies against their targets. Crystal structures of AmpC and RSK2 in complex with the new inhibitors confirmed the docking prediction's accuracy. For JAK3 these are the first reversible covalent molecules ever reported. The program can be accessed at <http://covalent.docking.org/>

**CovalentDock Cloud**

CovalentDock Cloud was developed by Ouyang et al. [41], it is a web interface to perform covalent docking experiments and analysis online based on the CovalentDock tool [17]. The web server accepts the structures of both the ligand and the receptor uploaded by the user or retrieved from online databases. It identifies the potential covalent binding patterns, carries out the covalent docking experiments, and provides visualization of the results for user analysis. This web server is available at <http://docking.sce.ntu.edu.sg/>.

**HCovDock**

HCovDock is a web server developed by Wu et al. [42] for investigating covalent protein-ligand interactions by integrating a ligand sampling method of incremental construction and a scoring function that takes into account covalent bond-based energy correction to reduce the unfavorable VDW energy for the ligand atoms in the region near the reactive residue. The incremental construction method is adapted (or modified) from UCSF DOCK 4 [76], in which two virtual atoms are added to the warhead for the ligand attached to the receptor by superimposing two virtual atoms of the ligand to the reactive residue of the receptor.

The program was benchmarked using 207 diverse protein-ligand complexes showing that HCovDock exhibits a significantly better performance than seven other state-of-the-art covalent docking programs (AutoDock, Cov_DOX, CovDock, FITTED, GOLD, ICM-Pro, and MOE) on this selected test set. With the criterion of ligand RMSD < 2.0Å, HCovDock obtained a high success rate of 70.5% and 93.2% in reproducing experimentally observed structures for the top 1 and top 10 predictions. In addition, HCovDock was also validated in VS against 10 receptors of three proteins (cSrc, EGFR, JNK). HCovDock is computationally efficient, and the average running time for docking a ligand is only 5 min with as fast as 1 sec for ligands with one rotatable bond and about 18 min for ligands with 23 rotational bonds. In a recent study, Weng et al. used HCovDock to explore the binding mode of a covalent inhibitor (OCM-1) at the ATP binding pocket of Bruton's tyrosine kinase (BTK). The covalent docking results showed that the core structures and covalent warheads of OCM-1 and ibrutinib share similar interactions with the BTK protein, supporting the irreversible covalent binding of OCM-1 with the thiol group of Cys481, leading to the inactivation of the BTK protein [77]. HCovDock can be freely assessed at <http://huanglab.phys.hust.edu.cn/hcovdock/>.

**SCARDock**

SCARdock is a free web server for screening covalent ligands of submitted proteins [43]. It is based on the Steric-Clashes Alleviating Receptor (SCAR) approach that screens covalent ligands based on noncovalent docking, and the method has been validated experimentally [78]. In the SCAR strategy, the covalent residue in the protein is mutated or removed before docking to eliminate the steric collisions from a covalent group. Then, a noncovalent docking process is performed for VS. The distance between the bonding atoms is used for evaluating the possibility of covalent bonding. Users need to designate the target protein by providing a PDB ID or uploading a PDB file directly, specify the targeting residue, and choose a ligand data set for docking. The server performs the docking process with AutoDock Vina v 1.1.2. The results are filtered according to the docking scores, score density, and the distances between the assumed covalent atoms. The server also provides three datasets useful for the discovery of covalent ligands:

1. A manually curated dataset containing 954 published high-quality complex structures of covalent ligands and protein targets.
2. A manually collected and curated dataset of 68 experimentally confirmed covalent warheads targeting 11 residues.
3. A pre-filtered and ready-to-use dataset containing 690,018 entries of purchasable compounds containing 68 warheads targeting 11 residues.

In a recent study, Zhang et al SCARdock performed the VS to identify covalent allosteric inhibitors of SUMO E1, a heterodimer formed by Aos1 (or SAE1) and Uba2 (or SAE2). In the first step, the covalent residue Cys30 was virtually mutated to glycine as per the SCARdock protocol. A pre-filtered dataset of 106,952 compounds from the MCULE screening library (https://mcule.com) based on manually curated warheads 23 was used for docking evaluation. Based on the lowest docking score, conformation ranking, covalent atom distance, and score density, 2,026 virtual hits were obtained for visual verification. After discarding hits with misaligned warheads and considering warhead diversity, 13 compounds with eight different warheads were experimentally validated. The experiments identified the covalent binding of two ligands to Uba2 [79]. These covalent allosteric inhibitors also affected the SUMOylation of the methionine adenosyltransferases MAT2.

In another study, Ai et al. used the enhanced version of the SCARdock protocol by incorporating quantum chemistry-based warhead reactivity calculations to identify 12 new S-adenosylmethionine decarboxylase (AdoMetDC) covalent inhibitors from 17 compounds, achieving a 70.6% hit rate [80]. The SCARdock web server can be accessed at <http://www.liugroup.site/scardock/> or <https://scardock.com/#/>.

**CovPepDock**

CovPepDock is a tool for designing peptide binders that form an irreversible covalent bond with a target cysteine residue, starting from a known non-covalent binder [44,45]. As input, the user should provide a PDB structure of a peptide-protein complex and specify the target cysteine and peptide chain. The protocol identifies all the peptide positions that are close to the target cysteine and mutates each of these positions to various electrophiles. It then performs docking simulations for each of these putative peptides, using the Rosetta FlexPepDock application [81,82] (<http://flexpepdock.furmanlab.cs.huji.ac.il/>), with the addition of constraints that favor the formation of a covalent bond between the electrophile and the target cysteine. As output, the protocol provides a list of the most promising peptide candidates with modifications suitable for irreversible binding. The candidates are selected based on their interface score, covalent constraint score, and backbone interface RMSD. The CovPepDock was applied retrospectively to a dataset of 115 disulfide-bound peptides and a dataset of 54 electrophilic peptides. It produced a top-five scoring, near-native model, in 89% and 100% of the cases when docking from the native conformation, and 20% and 90% when docking from an extended peptide conformation, respectively. In addition, a protocol was developed for designing electrophilic peptide binders based on known noncovalent binders or protein-protein interfaces. The study further identified 7154 peptide candidates in the PDB for the application of this protocol. As a proof-of-concept, the protocol was validated on the noncovalent complex of 14-3-3σ and YAP1 phosphopeptide. The protocol identified seven highly potent and selective irreversible peptide binders. The predicted binding mode of one of the peptides was validated using X-ray crystallography [83]. The CovPepDock server can be accessed at <https://rosie.rosettacommons.org/cov_pep_dock/>.

**HADDOCK 2.4**

HADDOCK (**H**igh **A**mbiguity **D**riven protein-protein **DOCK**ing) ( <https://www.bonvinlab.org/software/haddock2.4>) is an information-driven, flexible docking approach for the modeling of biomolecular complexes. The program is a collection of Python scripts derived from ARIA (<https://aria.pasteur.fr>) that utilize the experimental data of CNS (Crystallography and NMR System – <https://cns-online.org>) as restraints and use them to guide the docking process or structure calculation of molecular complexes alongside traditional energetics and shape complementarity [46–49]. The HADDOCK developer team defined a standard covalent docking protocol that was applied to a set of covalent inhibitors of cathepsin K [84]. The covalent bond between cysteine and the inhibitor is modeled by scaling down the van der Waals radius of the Cysteine sulfur atom 10-fold and introducing two distance restraints: (i) between the sulfur atom of the targeted cysteine and the reactive carbon atom of the ligand, set to 1.8 ± 0.1Å (i.e. the average length of a single C-S bond) and (ii) between the cysteine Cβ atom and the ligand carbon atom adjacent to the reactive carbon, set to 2.8 ± 0.1Å (i.e. the same as between the Cγ and Cϵ atoms of methionine, to model the proper angular geometry). The covalent docking is carried out in three different stages: (1) A rigid body docking is first performed with all geometrical parameters treated as fixed and allowing 180° rotations to generate 1,000 initial poses. After minimization, the best-scoring 200 poses are selected for further refinement. (2) A semi-flexible simulated annealing simulation (SA) in torsion angle space is applied to introduce flexibility to the interacting partners through a three-step MD-based refinement to optimize interface packing. The interacting partners are first kept rigid, and only their orientations are optimized. Flexibility is then introduced in the interface, which is automatically defined based on an analysis of intermolecular contacts within a 5Å cut-off. This allows different binding poses coming from the first stage of docking to have different flexible regions defined. Residues belonging to this interface region are then allowed to move their side chains in a second refinement step. Finally, both the backbone and side chains of the flexible interface are granted freedom. (3) The final stage of the docking protocol immerses the complex in a solvent shell to improve the energetics of the interaction. In this short explicit solvent refinement, the models are subjected to a short MD simulation at 300K, with position restraints on the non-interface heavy atoms. These restraints are later relaxed to allow all side chains to be optimized. The final optimized poses are automatically clustered based on a specific similarity measure - either the positional interface ligand RMSD (iL-RMSD) or the fraction of common contacts that measures the similarity of the intermolecular contacts. Throughout the whole protocol, HADDOCK’s scoring function for small molecule docking is applied. The HADDOCK web server can be accessed at <https://rascar.science.uu.nl/haddock2.4/> [85].

Due to the time-consuming steps, HADDOCK does not seem to be appropriate for performing VS. However, HADDOCK could be of great interest in understanding the binding of covalent compounds and their optimization. The covalent docking protocol seems very robust, but it does not consider the covalent bond energy contribution to the docking score.

In a recent study, Mueller et. al used the covalent docking protocol of HADDOCK to understand the molecular determinants of acrylamide reactivity with cysteines in proteins. In particular, authors focused on neuronal protein targets associated with neurotoxic symptoms of acrylamide, as well as acrylamide-modified proteins detectable in plasma and liver that can be used as biomarkers to monitor ACR exposure. Based on the analysis of the modeled acrylamide-protein complexes, the authors concluded that acrylamide modification is favored in the presence of nearby positively charged amino acids, such as lysine and arginine, and such a microenvironment facilitates the Michael addition reaction and stabilizes the resulting adduct. For proteins with more than one reactive Cys residue, the obtained docking scores were able to discriminate between the primary acrylamide binding site and secondary sites modified only at high acrylamide concentrations. Therefore, docking scores emerge as a potential filter to predict cysteine reactivity against acrylamide [86].

In another study, Anchoori et al used HADDOCK to model a covalent complex of Regulatory particle non-ATPase 13 (RPN13) Pru domain and the covalent ligand RA183. Four distance restraints were defined between Cys88 Sγ of RPN13 and four atoms of RA183 to recapitulate the sulfur–carbon bond. Ambiguous interaction restraints (AIRs) were imposed to restrict hRPN13 Pru active residues to be within 2.0Å of any RA183 atom. A total of 1000 predicted covalent complexes were subjected to rigid-body energy minimization, and 250 lowest-energy structures were chosen for semiflexible simulated annealing in torsion angle space, followed by refinement in explicit water. During semiflexible simulated annealing, atoms at the interface were allowed to move, but constrained by the different interactions and distance constraints defining the sulfur-carbon bond. After water refinement, 250 RPN13 Pru–RA183 structures were sorted into three clusters by using a 1.5Å cutoff criterion on the root-mean-square deviation of RA183. The lowest-energy structure of each cluster was energy-minimized by Schrödinger after the explicit introduction of a covalent bond between RPN13 Cys88 Sγ and the RA183 reacted carbon [87].

**DockThor-VS**

The DockThor-VS platform is a free protein-ligand docking server for performing docking-based VS experiments using high-performance computing [50–52]. The DockThor docking engine is a grid-based method designed for flexible-ligand and rigid-receptor docking. It employs a multiple-solution genetic algorithm and the MMFF94S molecular force field scoring function for pose prediction. Affinity prediction and ranking of protein-ligand complexes are performed with the linear empirical scoring function DockTScore [53]. The results of docking experiments are automatically clustered and ordered, providing users with a diverse array of binding modes. However, the DockThor program is not configured to perform covalent docking yet, it can predict the binding mode of the non-covalent receptor-ligand complex occurring in the pocket before the covalent bond formation. Therefore, the DockThor-VS web server could be useful as a fast tool to predict receptor-ligand binding modes to be investigated further for potential covalent-bonding formation using suitable methodologies. The DockThor-VS docking server can be accessed at <https://dockthor.lncc.br/v2/>.

**Pharmacophore-based tools for modeling covalent ligands**

**LigandScout**

LigandScout is a fully integrated commercial platform for computer-aided molecular design based on advanced and user-friendly pharmacophore modeling and VS [54–56]. A pharmacophore is defined as an ensemble of universal chemical features that characterize a specific mode of action of a ligand in the active site of the macromolecule in 3D space. Chemical features are, e.g., hydrogen bonds, charge interactions, and hydrophobic regions. This pharmacophoric pattern is the condition for ligand-protein interaction, and this can be used to search similar chemical patterns in large molecule databases to find new scaffolds for developing lead structures. Apart from modeling the noncovalent interaction, LigandScout can be customized to deal with covalent inhibitors by adding a chemical feature that represents the electrophilic point of the warhead.

For instance, Schulz et al. used LigandScout for the identification of covalently binding fragments of enteroviral 3C protease by implementing reactive groups potentially forming covalent bonds as a chemical feature in the 3D pharmacophore modeling program of LigandScout [88]. In the first step, covalently binding substructures were assembled from the literature. The covalently binding substructures, which are not applicable for rational design, were discarded. For the selected warheads, SMARTS patterns were developed and implemented in LigandScout. The covalent bond feature was represented by a sphere, which is placed on the ligand atom covalently bound or binding to the protein. From crystal structures of peptidic ligands covalently bound to the CVB3 3Cpro, a 3D pharmacophore model was generated, which comprises the essential noncovalent interactions and, in addition, reflects the necessary spatial positioning of the warhead moiety for covalent interaction with the catalytic Cys147 of the 3Cpro. The model was subsequently validated by using non-peptidic ligands reported in the literature and has a high specificity of 0.87 and sensitivity of 0.99. The generated 3D pharmacophore model was subsequently used to screen a set of fragment-like molecules. The screening resulted in 47 virtual hits, which were further analyzed through covalent and noncovalent docking into the 3Cpro active site. From the analysis of docking poses, 19 fragments representing 12 different chemotypes were selected that displayed a consistent 3D pharmacophore model. From each of these, one fragment was selected for biological evaluation in an enzyme kinetic assay for 3Cpro. Six fragments inhibited the 3Cpro by more than 30 % at 650 μM., with the best fragment showing an apparent IC_50_ of 355 ± 30 μM.

In a recent study, Soubhye et al. performed a novel VS procedure by combining ligand-based pharmacophore modeling and SBVS to discover reversible and irreversible inhibitors of the heme enzyme myeloperoxidase (MPO) [89]. In the first step, four pharmacophore models were designed based on the most potent MPO inhibitors using LigandScout. These pharmacophore models were used to screen a filtered Zinc database. Compounds selected from this VS were docked into the heme cavity of MPO utilizing the LeadIT docking program [90] to screen the Zinc database [91] for potential ligands. 28 molecules were selected by this virtual method and tested on MPO in vitro. Twelve out of 28 compounds were found to have an IC_50_ less than 5 μM. The best inhibitors showed IC50 values of 44 and 50 nM, respectively. Studies on the mechanism of inhibition indicated that one of the compounds inhibits MPO irreversibly at a nanomolar concentration. The Ligandscout software is available at <https://www.inteligand.com/ligandscout/>.

**AncPhore**

AncPhore is a pharmacophore-based tool for drug discovery, which is characterized by pharmacophore feature analysis and anchor pharmacophore (i.e., the most important pharmacophore features) steered molecular fitting and VS [57]. AncPhore involves ten types of pharmacophore features, including hydrogen bond donor (HD), hydrogen bond acceptor (HA), positively charged center (PO), negatively charged center (NE), metal coordination (MB), halogen bonding (XB), aromatic ring (AR), cation-interaction (CR), hydrophobic (HY), and covalent bonding (CV) feature. The exclusion spheres are also included (EX). AncPhore provides a framework to generate a pharmacophore model for a ligand structure, an apo-protein structure, or a protein-ligand complex structure, and to perform VS, target profiling, drug repositioning, and so on. AncPhore presently supports four main types of covalent-bonding modes, and reactive groups for ligands known to bind cysteine (−SH), serine/threonine/tyrosine (−OH), lysine (−NH2), or glutamic acid/aspartic acid (−COOH). The covalent bond features are defined according to the distance between protein and ligand reactive atoms without consideration of the bond direction. In an apo protein structure, the CV feature is generated for specific residues, such as cysteine, serine, threonine, and lysine, which is positioned in specific environments to form covalent bonds with small molecules. Other standard pharmacophore features, such as HA, HD, PO, NE, and HY are analyzed based on molecular interaction fields (ID-Score) [92], and the k-means clustering technique. The AncPhore is free for academic use, and the tool can be downloaded at <https://ancphore.ddtmlab.org/>.

**CSD-CrossMiner**

CSD-CrossMiner is a commercial pharmacophore modeling tool of CCDC that allows users to build 3D pharmacophore queries describing protein-ligand interactions, ligand scaffolds, or protein environments and modify those queries interactively [58]. The pharmacophore query can be used to mine structures that match the Cambridge Structural Database (CSD), the Protein Data Bank (PDB), and any in-house structural databases. Applications of CSD-CrossMiner include finding bioisosteres, evaluating potential off-target effects of drug molecules by comparing binding sites, and searching for new scaffolds to improve the pharmacokinetic properties of an existing bioactive compound. CSD-CrossMiner includes several diverse pharmacophore features: Features: heavy_atom, hydrophobic, acceptor, acceptor_projected, donor_projected, donor_ch_projected, planar_ring_projected, ring_projected, water, exit_vector, halogen, and metal. An excluded volume for a ligand in a protein−ligand binding site can be defined by a protein “heavy_atom” feature at the desired excluded position. Besides studying pharmacophores for numerous noncovalent protein-ligand complexes of different targets, the tool was also applied to study inhibitors of the cysteine protease cathepsin L that typically form a covalent bond with the active site Cys25 and gain additional binding affinity by occupying the S2 and S3 pockets [93–96]. To explore new covalent inhibitors for different residue types, it is possible to annotate electrophilic warheads within CSD-CrossMiner through substructure-based features that are defined by a hierarchy of SMARTS patterns[97] for performing search. The CSD-CrossMiner can be obtained at <https://www.ccdc.cam.ac.uk/solutions/software/csd-crossminer/>.

**Phase**

Phase is a versatile and easy-to-use pharmacophore modeling tool from Schrödinger that allows the assessment of compounds based on the steric and electronic features of molecules known to have biological activity. Phase employs a unique common pharmacophore perception algorithm designed for use in both lead optimization and VS, and helps create an understanding of an unknown binding site in the absence of a protein structure. The default features are hydrogen bond donor (D) and acceptor (A), aromatic ring (R), hydrophobic (H), as well as positive (P) and negative (N) ionic. Pharmacophore models can be developed from a single ligand, a set of ligands, a ligand-receptor complex, or an apo-protein. Custom features such as covalent bonding and geometric constraints on distances, angles, and dihedrals can be added to a hypothesis for a ligand to match with a hypothesis [59,60]. The details on the Phase pharmacophore modeling software are available at <https://www.schrodinger.com/platform/products/phase/>.

**Catalyst**

Catalyst develops 3D models called hypotheses from a collection of molecules possessing a range of diversity in both structures and activities. One can use these hypotheses as queries to search 3D databases to retrieve structures that fit the hypothesis, or as models to predict the activities of novel compounds. Typically, pharmacophore features consist of a hydrogen bond acceptor, hydrogen bond donor, hydrophobic, negative charge, positive charge, and aromatic ring. It is possible to add a predefined pharmacophore feature, such as covalent bonding, and make modifications [61]. The catalyst pharmacophore modeling tool is now integrated into the Biovia Discovery studio software, and the details on the software are available at <https://www.3ds.com/products/biovia/discovery-studio/ligand-and-pharmacophore-based-design>.

**Databases of covalent ligands and 3D covalent protein-ligand complexes**

**Cysteinome**

Cysteinome is the first online database for performing search and analysis of structure, function, and related annotation for proteins with targetable cysteine as well as their covalent modulators curated from literatures [62]. The database has 462 proteins with targetable cysteine residues from 122 different species, along with 1217 covalent modulators. Proteins are annotated with a detailed description of protein families, biological processes, and related diseases. In addition, covalent modulators are annotated with chemical names, chemical structure, binding affinity, physicochemical properties, molecule type, related diseases, etc. The database could help medicinal chemists in the design and discovery of covalent inhibitors targeting functional cysteines of critical protein targets implicated in various physiological or disease processes. The Cysteinome database is now available at <http://www.cysteinome.net/Cysteinome/index.php/Cysteinome>.

**cBinderDB**

cBinderDB is a database providing information on covalent binding compound structures, chemotypes, targets, covalent binding types, and other biological properties [98]. The covalent binding targets are annotated with biological functions, protein family and domains, gene information, modulators, and receptor-ligand complex structure. The authors compiled the data from different scientific publications (from 1960 up to 2016) by combining a text-mining method and manual inspection processes. In total, there are 527 covalent binders and 190 related protein targets within the cBinderDB. More than 95% of covalent binders are inhibitors against their targets, others are activators or modulators. cBinderDB is freely available at [www.rcdd.org.cn/cbinderdb/](http://www.rcdd.org.cn/cbinderdb/) (for the time being the website is no longer active).

**CovalentInDB 2.0**

CovalentInDB (Covalent Inhibitor Database) 2.0 is an updated version of the CovalentInDB database [99] designed to support covalent drug discovery [63]. It is the largest web-accessible resource for covalent inhibitors and related targets. It provides not only basic information about covalent inhibitors, targets, and bioactivity but also the reactive warheads of inhibitors, covalent reaction mechanisms, attacked nucleophilic residues, and covalent-mechanism experimental verification methods. The latest version of the database (2025) includes 8303 covalent inhibitors and 368 targets, supplemented by 3445 newly added cocrystal structures, providing detailed analyses of noncovalent interactions. To expand the research of covalent ligands to the proteome level, we profiled the entire structures in the RCSB Protein Data Bank (PDB) with a well-validated AI model (DeepCoSI) to evaluate the ligandability of 144,864 cysteines across the human proteome for covalent ligand design. DeepCoSI is a structure-based Deep Graph Learning Network Method for Covalent Binding Site Identification [100]. The codes for DeepCoSI are available at <https://github.com/Brian-hongyan/DeepCoSI>. CovalentInDB 2.0 also features the largest covalent VS library with 2,030,192 commercially available compounds and a natural product library with 105,901 molecules, crucial for covalent drug screening and discovery. To enhance the utility of these compounds, the authors performed structural similarity analysis and drug-likeness predictions. Additionally, a new user data upload feature enables efficient data contribution and continuous updates. A molecular visualization function is also embedded to display protein-ligand interaction. CovalentInDB 2.0 is freely accessible at <http://cadd.zju.edu.cn/cidb/>. The database could provide useful information for the structure-based rational design of targeted covalent inhibitors.

**CovBinderInPDB**

Guo and Zhang developed an integrated computational protocol to curate covalent binders from the RCSB Protein Data Bank (PDB) [64]. Starting from the macromolecular crystallographic information files (mmCIF) in the PDB archive, covalent bond records, which indicate the side chain modification of the amino acid residue by a covalent binder, were collected and cleaned. Then, residue–binder adducts, which are products of chemical reactions between targeted residues and covalent binders, were recovered with the help of the Chemical Component Dictionary in PDB. Finally, several strategies were employed to curate the pre-reaction forms of covalent binders from the adduct. The database contains 7375 covalent modifications in which 2189 unique covalent binders target nine types of amino acid residues (Cys, Lys, Ser, Asp, Glu, His, Met, Thr, and Tyr) from 3555 complex structures of 1170 unique protein chains. The database could help develop and benchmark computational strategies for covalent inhibitor design and is freely accessible at <https://yzhang.hpc.nyu.edu/CovBinderInPDB>.

**CovPDB**

CovPDB is the first database solely devoted to high-resolution cocrystal structures of biologically relevant covalent protein-ligand complexes, curated from the Protein Data Bank (PDB) [65]. For these curated complexes, the chemical structures and warheads of pre-reactive electrophilic ligands, as well as the covalent bonding mechanisms to their target proteins, were manually annotated. In total, CovPDB contains 733 proteins and 1,501 ligands, relating to 2,294 covalent complexes, 93 reactive warheads, 14 targetable residues, and 21 covalent binding mechanisms. Users are provided with an interactive web interface that allows multiple search and browsing options to explore the covalent interactome at a molecular level to develop novel TCIs. CovPDB is freely accessible at <http://www.pharmbioinf.uni-freiburg.de/covpdb/>.

**Cysteine reactivity prediction tools**

**CovCys**

CovCys was developed based on a comprehensive statistical analysis of covalently modified cysteine residues in protein structures by Zhang et al. An SVM-based machine-learning technique was applied to a large dataset of protein-ligand complexes with covalent ligands attached to cysteine residues [101]. The study showed that covalent-modified cysteine residues have unique features compared to those not attached to covalent ligands, including lower pKa, higher exposure, and higher ligand binding affinity. The SVM models were able to correctly predict cysteine residues suitable for covalent ligand design with a prediction accuracy of 0.73. Given a protein structure, this method can be used to automatically detect a novel binding site containing druggable cysteine residues for covalent ligand design. The CovCys tool is integrated within CavityPlus, a web server for protein cavity detection with pharmacophore modeling, allosteric site identification, and covalent ligand binding ability prediction [102]. The CovCys can be accessed at <http://www.pkumdl.cn:8000/cavityplus/index.php#/>.

**SILCS-Covalent**

In a recent study, Yu et al. applied the site identification by ligand competitive saturation (SILCS) method, SILCs-Covalent, for identifying reactive cysteines that can react with electrophilic probes [103]. The SILCS method [104] is a functional group mapping approach using explicit solvent all-atom oscillating excess chemical potential, µex, Grand Canonical Monte Carlo (GCMC)/MD simulations [105] to model the interactions of selected probe solute molecules as well as water with a target protein. Through docking of a library of representative warhead fragments using SILCS-Monte Carlo (SILCS-MC), reactive cysteines were correctly identified for proteins being tested. Furthermore, the authors trained a machine learning model to quantify the effectiveness of various warhead groups for proteins using metrics from SILCS-MC as well as experimental model compound warhead reactivity data. The ability to rank covalent molecular binders with similar warheads using SILCS ligand grid free energy (LGFE) ranking was also tested for several proteins. Despite being very computationally expensive, the SILCS-Covalent approach can be more accurate in identifying accessible and reactive sites.

**ICM**

The ICM package has a tool that can predict the reactivity of a Cys residue. The method is based on reactivity data for 34 reactive and 184 non-reactive cysteines from isoTOP-ABPP (isotopic tandem orthogonal proteolysis activity-based protein profiling) [106], and a nonredundant set of PDB protein structures. For each of the cysteine residues analyzed the program outputs the distance of the residue to the protein surface, the solvent accessible surface area of the Cys side chain, the reactivity score (the higher the more reactive), pocket area (if there is a binding pocket within 2.5A of a CYS residue), - distance to nearest positively charged Lys and Arg residues, distance to nearest Hip/His/Hie, the electrostatic potential at S(Beta) of a Cys, and energy of hydrogen bonds with backbone [10]. The ICM software can be downloaded at <https://molsoft.com/products.html>.

**Cpipe**

Cpipe is a computational platform for the Identification and classification of reactive cysteines. The program performs the reactivity analysis, evaluation of the structure-based features (to assess intrinsic Cys reactivity), and the functional analysis (prediction of functional roles: metal binding, disulfide, catalytic, and post-translational modifications) [107]. Cpipe is implemented in Python and is freely available at <http://cpipe.explora-biotech.com/cpipe/start.py>. (NB: on july 2025, page not available)

**BioLuminate**

Schrodinger’s BioLuminate is a commercial tool that can identify reactive residues by matching residue patterns in the sequence. Six patterns are used that represent the common reactions: Deamidation, Oxidation, Glycosylation, Isomerization, Free Cysteine, and Proteolysis. The reactive sequences are also matched to extended PROSITE patterns that define the reactive sites. In the end, the calculations provide the type of reactive site, Reactive residue identity, Solvent-accessible surface area of the residue, Solvent exposure of the residue (percentage of the surface that is solvent-exposed), and PDB temperature factor for the residue, if available [108–110]. Schrodinger’s BioLuminate tool is available at <https://www.schrodinger.com/platform/products/bioluminate/>.

**HyperCys**

HyperCys is a novel ensemble stacked machine learning (ML) model to predict hyper-reactive druggable cysteines [111]. First, the pocket, conservation, structural, and energy profiles, and physicochemical properties of (non)covalently bound cysteines were collected from both protein sequences and 3D structures of protein-ligand complexes. Then, the authors established the HyperCys ensemble stacked model by integrating six different ML models, including K-nearest neighbors, support vector machine, light gradient boost machine, multi-layer perceptron classifier, random forest, and the meta-classifier model logistic regression. Finally, based on the hyper-reactive cysteines’ classification accuracy and other metrics, the results for different feature group combinations were compared. The HyperCys was able to achieve the accuracy, F1 score, recall score, and ROC AUC values of HyperCys of 0.784, 0.754, 0.742, and 0.824, respectively, after performing a 10-fold CV with the best window size. Compared to traditional ML models with only sequence-based features or only 3D structural features, HyperCys was found to be more accurate at predicting hyper-reactive druggable cysteines. HyperCys can be an effective tool for discovering new potential reactive cysteines for a wide range of targeted proteins.

**Tools for identifying novel reactive ligands**

Analogous to the identification of reactive residues in the binding sites that can serve as potential nucleophiles, some recent studies have put efforts into identifying electrophilic ligands from a large database of small molecules that can react with proteins. For instance, Gil and Rowley et al. employed traditional and graph machine learning (ML) algorithms to classify molecules as reactive or nonreactive towards proteins. For training, a dataset, ProteinReactiveDB, was built that was composed primarily of covalent and noncovalent inhibitors from the DrugBank [112], BindingDB [113], and CovalentInDB [99] databases. To assess the transferability of the trained models, the authors created a custom set of covalent and noncovalent inhibitors from the recent literature. Among all models, Graph Neural Networks (GNNs) showed the best results with an Area Under the Receiver Operator Characteristic (AUROC) curve of 0.80, precision of 0.89, and recall of 0.72. The study further explored the interpretability of these GNNs using Gradient Activation Mapping (GradCAM), which shows regions of the molecules GNNs deem most relevant when making a prediction. These maps indicated that the trained models can identify electrophilic functional groups in a molecule and classify molecules as protein-reactive based on their presence. To demonstrate the use of these models, the authors compared the performance of the models against common chemical filters, identified covalent modifiers in the ChEMBL database [114], and generated a putative covalent inhibitor based on an established non-covalent inhibitor.

**ADMET of covalent ligands**

The formation of covalent bonds can significantly alter a compound’s behavior during absorption, distribution, metabolism, and excretion (ADME), potentially increasing the risk of toxicity. Covalent drugs may bind off-targets, from metabolic enzymes to plasma proteins, structural proteins, or membrane transporters, leading to altered distribution and potential bioaccumulation, like, for example, when the covalent binders or reactive metabolites of these compounds interact with lipids in specific tissues [115]. These interactions are often organ-specific and may result in long-term toxicity.

One of the most studied forms of toxicity is drug-induced liver injury (DILI), where covalent binding to metabolic enzymes (such as cytochrome P450s, Glutathion S-Transferases, NADPH-oxidases, Aminotransferases, Acyl CoA oxidases, Cyclooxygenases) or transporters can impair enzyme activity or provoke adaptive overexpression, disrupting metabolic homeostasis [116–118]. Inactivation of these enzymes not only contributes to DILI but also increases the risk of drug-drug interactions (DDIs) by altering the metabolism of co-administered therapies [119]. Enzyme inhibition by covalent binders induced non-reversible time-dependent inhibition processes where inhibition increases with time. For example, if a covalent drug irreversibly inhibits a major metabolic enzyme, it can lead to the accumulation of other drugs metabolized by the same pathway, potentially causing toxicity or therapeutic failure. It is important to emphasize, however, that both DILI and DDIs are multifactorial phenomena [120]. While the formation of reactive metabolites and irreversible enzyme binding are key contributors, other mechanisms, such as immune-mediated responses, transport inhibition, and genetic variability in drug metabolism, also play significant roles.

Covalent drugs typically undergo a two-step mechanism: an initial reversible binding to a nucleophilic site, followed by an irreversible covalent bond formation. This covalent attachment is often mediated by electrophilic warheads designed to react with specific nucleophilic residues such as cysteine, lysine, serine, or tyrosine. Common warheads include:

- Acrylamides: Form bonds with cysteine thiols through Michael addition
- Vinylsulfonamides: React with cysteines via Michael addition
- α,β-unsaturated carbonyls: Various Michael acceptors
- Epoxides: Ring-opening reactions with nucleophilic residues
- Boronic acids: Form reversible covalent bonds with serine residues
- Aldehydes: Form Schiff bases with lysine residues
- Activated esters: Acylate nucleophilic residues

In addition to direct covalent ligands, toxicity can also arise from metabolic activation, whereby the parent drug is transformed into a reactive intermediate capable of nonspecific covalent binding. These reactive metabolites, often formed in the active site of metabolic enzymes, can bind to proteins or DNA through mechanisms such as Michael addition, Schiff base formation, or nucleophilic substitution [121,122]^,^[123]^,^[124]^,^[125,126]. If these metabolites are highly reactive, they may be sequestered within the enzyme’s active site, leading to enzyme inactivation and numerous potential downstream effects [127]^,^ [128].

To mitigate damage, cells detoxify reactive metabolites using glutathione (GSH), which forms conjugates that facilitate excretion [129]. However, excessive generation of reactive species can deplete GSH reserves, resulting in oxidative stress, mitochondrial dysfunction, and mutagenic or immunogenic consequences.

Overall, the key types of toxicity associated with covalent drugs include:

-Off-target protein modification: disrupting normal protein function.

-Immunogenicity: neoantigen formation and hypersensitivity reactions.

-Glutathione depletion and oxidative stress (especially in the liver and CNS).

-Reactive metabolite formation: leading to hepatotoxicity or DILI.

-DNA binding/genotoxicity: increasing risk of mutagenesis or cancer.

-Mitochondrial toxicity: energy disruption and apoptosis.

Given these risks, various experimental and in silico tools have been developed to anticipate covalent liabilities [130]. While traditionally used in regulatory toxicology, assays for adduct formation and metabolic reactivity are increasingly applied in early drug discovery [131]. Nevertheless, these approaches remain resource-intensive and poorly suited for high-throughput screening. As a result, in silico models have emerged as valuable alternatives to flag potential risks earlier in the pipeline. Current strategies include:

- Fragment-based alerts and filtering (e.g., structural alerts [132], toxicophores [133], PAINS [134]),
- Indirect predictions where effects induced by the formation of covalent bonds are predicted (e.g., likelihood of DILI based on known liabilities [135] among many other types of adverse drug reaction prediction tools [136]),
- Metabolite structure generation and covalent binding prediction (various tools can be used from more traditional methods up to AI engines [137]^,^[138,139])
- Direct modeling of reactive metabolite formation [140], and
- Combined predictive frameworks designed to help medicinal chemists reduce adverse effect risks [141–143].

However, these computational approaches remain largely qualitative. They often predict relative, not absolute, metabolite abundances, and lack the resolution to model complete metabolic pathways across species or tissues. Current models cannot reliably quantify dose-exposure relationships or define thresholds for clinical relevance, and therefore should be interpreted as early warnings rather than definitive risk assessments. In addition, it is important to keep in mind that covalent binder identification with in silico tools allows to rank compounds on the same target, but not different targets for the same compound, without inter-target calibration protocols based on experimental data. This is particularly true when looking for specific covalent binders without ubiquitous covalent binding.

**References**

1. Sotriffer C. Docking of Covalent Ligands: Challenges and Approaches. Molecular Informatics 2018; 37:1800062

2. Strelow JM. A Perspective on the Kinetics of Covalent and Irreversible Inhibition. SLAS Discov 2017; 22:3–20

3. Zhao Z, Bourne PE. Advances in reversible covalent kinase inhibitors. Medicinal Research Reviews 2025; 45:629–653

4. Raouf YS. Covalent Inhibitors: To Infinity and Beyond. J. Med. Chem. 2024; 67:10513–10516

5. Bradshaw JM, McFarland JM, Paavilainen VO, et al. Prolonged and tunable residence time using reversible covalent kinase inhibitors. Nat Chem Biol 2015; 11:525–531

6. Jones G, Willett P, Glen RC, et al. Development and validation of a genetic algorithm for flexible docking. J. Mol. Biol. 1997; 267:727–748

7. Jones G, Willett P, Glen RC. Molecular recognition of receptor sites using a genetic algorithm with a description of desolvation. J. Mol. Biol. 1995; 245:43–53

8. Verdonk ML, Cole JC, Hartshorn MJ, et al. Improved protein-ligand docking using GOLD. Proteins 2003; 52:609–623

9. Scholz C, Knorr S, Hamacher K, et al. DOCKTITE-a highly versatile step-by-step workflow for covalent docking and virtual screening in the molecular operating environment. J Chem Inf Model 2015; 55:398–406

10. Abagyan R, Totrov M, Kuznetsov D. ICM—A new method for protein modeling and design: Applications to docking and structure prediction from the distorted native conformation. Journal of Computational Chemistry 1994; 15:488–506

11. Kramer B, Rarey M, Lengauer T. Evaluation of the FLEXX incremental construction algorithm for protein-ligand docking. Proteins 1999; 37:228–241

12. Cross SSJ. Improved FlexX Docking Using FlexS-Determined Base Fragment Placement. J. Chem. Inf. Model. 2005; 45:993–1001

13. Rarey M, Kramer B, Lengauer T, et al. A fast flexible docking method using an incremental construction algorithm. J. Mol. Biol. 1996; 261:470–489

14. Flare, version 6.0.; Cresset: Peterborough, UK, 2023; Available online: https://www.cresset-group.com/software/flare/.

15. Fradera X, Kaur J, Mestres J. Unsupervised guided docking of covalently bound ligands. J Comput Aided Mol Des 2004; 18:635–650

16. Fradera X, Knegtel RM, Mestres J. Similarity-driven flexible ligand docking. Proteins 2000; 40:623–636

17. Ouyang X, Zhou S, Su CTT, et al. CovalentDock: automated covalent docking with parameterized covalent linkage energy estimation and molecular geometry constraints. J Comput Chem 2013; 34:326–336

18. Bianco G, Forli S, Goodsell DS, et al. Covalent docking using autodock: Two‐point attractor and flexible side chain methods. Protein Sci 2016; 25:295–301

19. Morris GM, Huey R, Lindstrom W, et al. AutoDock4 and AutoDockTools4: Automated docking with selective receptor flexibility. J Comput Chem 2009; 30:2785–2791

20. McNutt AT, Li Y, Meli R, et al. GNINA 1.3: the next increment in molecular docking with deep learning. Journal of Cheminformatics 2025; 17:28

21. Tan YS, Chakrabarti M, Stein RM, et al. Development of Receptor Desolvation Scoring and Covalent Sampling in DOCK 6: Methods Evaluated on a RAS Test Set. J Chem Inf Model 2025; 65:722–748

22. Wu Y, Brooks Iii CL. Covalent docking in CDOCKER. J Comput Aided Mol Des 2022; 36:563–574

23. Hu Q, Wang Z, Meng J, et al. OpenDock: a pytorch-based open-source framework for protein–ligand docking and modelling. Bioinformatics 2024; 40:btae628

24. Wei L, Wen W, Rao L, et al. Cov_FB3D: A De Novo Covalent Drug Design Protocol Integrating the BA-SAMP Strategy and Machine-Learning-Based Synthetic Tractability Evaluation. J Chem Inf Model 2020; 60:4388–4402

25. Rachman M, Scarpino A, Bajusz D, et al. DUckCov: a Dynamic Undocking-Based Virtual Screening Protocol for Covalent Binders. ChemMedChem 2019; 14:1011–1021

26. Zhu K, Borrelli KW, Greenwood JR, et al. Docking covalent inhibitors: a parameter free approach to pose prediction and scoring. J Chem Inf Model 2014; 54:1932–1940

27. Toledo Warshaviak D, Golan G, Borrelli KW, et al. Structure-based virtual screening approach for discovery of covalently bound ligands. J Chem Inf Model 2014; 54:1941–1950

28. Goullieux M, Zoete V, Röhrig UF. Two-Step Covalent Docking with Attracting Cavities. J Chem Inf Model 2023; 63:7847–7859

29. Scarpino A, Petri L, Knez D, et al. WIDOCK: a reactive docking protocol for virtual screening of covalent inhibitors. J Comput Aided Mol Des 2021; 35:223–244

30. Corbeil CR, Englebienne P, Moitessier N. Docking ligands into flexible and solvated macromolecules. 1. Development and validation of FITTED 1.0. J Chem Inf Model 2007; 47:435–449

31. Corbeil CR, Englebienne P, Yannopoulos CG, et al. Docking ligands into flexible and solvated macromolecules. 2. Development and application of fitted 1.5 to the virtual screening of potential HCV polymerase inhibitors. J Chem Inf Model 2008; 48:902–909

32. Moitessier N, Pottel J, Therrien E, et al. Medicinal Chemistry Projects Requiring Imaginative Structure-Based Drug Design Methods. Acc. Chem. Res. 2016; 49:1646–1657

33. Warrensford L, Pittman AR, Austin S, et al. CovCIFDock: Covalent Docking with CIFDock and Hybrid QM/MM Minimizations. 2025;

34. Shamir Y, London N. State-of-the-art covalent virtual screening with AlphaFold3. 2025; 2025.03.19.642201

35. Shen C, Du H, Zhang X, et al. CarsiDock-Cov: A deep learning-guided approach for automated covalent docking and screening. Acta Pharmaceutica Sinica B 2025;

36. Peng Y, Gao K, He L, et al. CovDocker: Benchmarking Covalent Drug Design with Tasks, Datasets, and Solutions. Proceedings of the 31st ACM SIGKDD Conference on Knowledge Discovery and Data Mining V.2 2025; 2281–2290

37. Lai H, Wang L, Qian R, et al. Interformer: an interaction-aware model for protein-ligand docking and affinity prediction. Nat Commun 2024; 15:10223

38. Wei L, Chen Y, Liu J, et al. Cov_DOX: A Method for Structure Prediction of Covalent Protein-Ligand Bindings. J Med Chem 2022; 65:5528–5538

39. Zhou Y, Yu H, Vind AC, et al. Rational Design of Covalent Kinase Inhibitors by an Integrated Computational Workflow (Kin-Cov). J Med Chem 2023; 66:7405–7420

40. London N, Miller RM, Krishnan S, et al. Covalent docking of large libraries for the discovery of chemical probes. Nat Chem Biol 2014; 10:1066–1072

41. Ouyang X, Zhou S, Ge Z, et al. CovalentDock Cloud: a web server for automated covalent docking. Nucleic Acids Res 2013; 41:W329-332

42. Wu Q, Huang S-Y. HCovDock: an efficient docking method for modeling covalent protein-ligand interactions. Brief Bioinform 2023; 24:bbac559

43. Song Q, Zeng L, Zheng Q, et al. SCARdock: A Web Server and Manually Curated Resource for Discovering Covalent Ligands. ACS Omega 2023; 8:10397–10402

44. Lyskov S, Chou F-C, Conchúir SÓ, et al. Serverification of molecular modeling applications: the Rosetta Online Server that Includes Everyone (ROSIE). PLoS One 2013; 8:e63906

45. Moretti R, Lyskov S, Das R, et al. Web-accessible molecular modeling with Rosetta: The Rosetta Online Server that Includes Everyone (ROSIE). Protein Sci 2018; 27:259–268

46. de Vries SJ, van Dijk M, Bonvin AMJJ. The HADDOCK web server for data-driven biomolecular docking. Nat Protoc 2010; 5:883–897

47. Dominguez C, Boelens R, Bonvin AMJJ. HADDOCK: a protein-protein docking approach based on biochemical or biophysical information. J Am Chem Soc 2003; 125:1731–1737

48. Kastritis PL, Rodrigues JPGLM, Bonvin AMJJ. HADDOCK(2P2I): a biophysical model for predicting the binding affinity of protein-protein interaction inhibitors. J Chem Inf Model 2014; 54:826–836

49. van Zundert GCP, Rodrigues JPGLM, Trellet M, et al. The HADDOCK2.2 Web Server: User-Friendly Integrative Modeling of Biomolecular Complexes. J Mol Biol 2016; 428:720–725

50. Guedes IA, Pereira da Silva MM, Galheigo M, et al. DockThor-VS: A Free Platform for Receptor-Ligand Virtual Screening. J Mol Biol 2024; 436:168548

51. Guedes IA, Costa LSC, Dos Santos KB, et al. Drug design and repurposing with DockThor-VS web server focusing on SARS-CoV-2 therapeutic targets and their non-synonym variants. Sci Rep 2021; 11:5543

52. Santos KB, Guedes IA, Karl ALM, et al. Highly Flexible Ligand Docking: Benchmarking of the DockThor Program on the LEADS-PEP Protein-Peptide Data Set. J Chem Inf Model 2020; 60:667–683

53. Guedes IA, Barreto AMS, Marinho D, et al. New machine learning and physics-based scoring functions for drug discovery. Sci Rep 2021; 11:3198

54. Wolber G, Langer T. LigandScout:  3-D Pharmacophores Derived from Protein-Bound Ligands and Their Use as Virtual Screening Filters. J. Chem. Inf. Model. 2005; 45:160–169

55. Wieder M, Garon A, Perricone U, et al. Common Hits Approach: Combining Pharmacophore Modeling and Molecular Dynamics Simulations. J. Chem. Inf. Model. 2017; 57:365–385

56. Wieder M, Perricone U, Boresch S, et al. Evaluating the stability of pharmacophore features using molecular dynamics simulations. Biochem. Biophys. Res. Commun. 2016; 470:685–689

57. Dai Q, Yan Y, Ning X, et al. AncPhore: A versatile tool for anchor pharmacophore steered drug discovery with applications in discovery of new inhibitors targeting metallo-β-lactamases and indoleamine/tryptophan 2,3-dioxygenases. Acta Pharm Sin B 2021; 11:1931–1946

58. Korb O, Kuhn B, Hert J, et al. Interactive and Versatile Navigation of Structural Databases. J Med Chem 2016; 59:4257–4266

59. Dixon SL, Smondyrev AM, Knoll EH, et al. PHASE: a new engine for pharmacophore perception, 3D QSAR model development, and 3D database screening: 1. Methodology and preliminary results. J Comput Aided Mol Des 2006; 20:647–671

60. Dixon SL, Smondyrev AM, Rao SN. PHASE: A Novel Approach to Pharmacophore Modeling and 3D Database Searching. Chemical Biology & Drug Design 2006; 67:370–372

61. CATALYST Pharmacophore Models and Their Utility As Queries for Searching 3D Databases - Computer‐Assisted Lead Finding and Optimization - Wiley Online Library.

62. Wu S, Luo Howard H, Wang H, et al. Cysteinome: The first comprehensive database for proteins with targetable cysteine and their covalent inhibitors. Biochem Biophys Res Commun 2016; 478:1268–1273

63. Du H, Zhang X, Wu Z, et al. CovalentInDB 2.0: an updated comprehensive database for structure-based and ligand-based covalent inhibitor design and screening. Nucleic Acids Research 2025; 53:D1322–D1327

64. Guo X-K, Zhang Y. CovBinderInPDB: A Structure-Based Covalent Binder Database. J Chem Inf Model 2022; 62:6057–6068

65. Gao M, Moumbock AFA, Qaseem A, et al. CovPDB: a high-resolution coverage of the covalent protein-ligand interactome. Nucleic Acids Res 2022; 50:D445–D450

66. Scarpino A, Ferenczy GG, Keserű GM. Comparative Evaluation of Covalent Docking Tools. J. Chem. Inf. Model. 2018; 58:1441–1458

67. Wen C, Yan X, Gu Q, et al. Systematic Studies on the Protocol and Criteria for Selecting a Covalent Docking Tool. Molecules 2019; 24:2183

68. Rao L, Chi B, Ren Y, et al. DOX: A new computational protocol for accurate prediction of the protein-ligand binding structures. J Comput Chem 2016; 37:336–344

69. Wei L, Chi B, Ren Y, et al. Conformation Search Across Multiple-Level Potential-Energy Surfaces (CSAMP): A Strategy for Accurate Prediction of Protein-Ligand Binding Structures. J Chem Theory Comput 2019; 15:4264–4279

70. Tsallis C, Stariolo DA. Generalized simulated annealing. Physica A: Statistical Mechanics and its Applications 1996; 233:395–406

71. Stewart JJP. Optimization of parameters for semiempirical methods VI: more modifications to the NDDO approximations and re-optimization of parameters. J Mol Model 2013; 19:1–32

72. Guo W, Wu A, Zhang IY, et al. XO: an extended ONIOM method for accurate and efficient modeling of large systems. J Comput Chem 2012; 33:2142–2160

73. Cao H, Huang Z, Liu Z, et al. Structure-Guided Design of Affinity/Covalent-Bond Dual-Driven Inhibitors Targeting the AMP Site of FBPase. J Med Chem 2024; 67:20421–20437

74. Bianco G, Holcomb M, Santos-Martins D, et al. Reactive Docking: A Computational Method for High-Throughput Virtual Screenings of Reactive Species. J Chem Inf Model 2023; 63:5631–5640

75. Mysinger MM, Shoichet BK. Rapid Context-Dependent Ligand Desolvation in Molecular Docking. J. Chem. Inf. Model. 2010; 50:1561–1573

76. Ewing TJ, Makino S, Skillman AG, et al. DOCK 4.0: search strategies for automated molecular docking of flexible molecule databases. J. Comput. Aided Mol. Des. 2001; 15:411–428

77. Weng W, Zhang P, Pan Z. Potent Inhibition and Rapid Photoactivation of Endogenous Bruton’s Tyrosine Kinase Activity in Native Cells via Opto-Covalent Modulators. J Am Chem Soc 2024; 146:28717–28727

78. Ai Y, Yu L, Tan X, et al. Discovery of Covalent Ligands via Noncovalent Docking by Dissecting Covalent Docking Based on a ‘Steric-Clashes Alleviating Receptor (SCAR)’ Strategy. J Chem Inf Model 2016; 56:1563–1575

79. Zhang S, Wang Z, Jiao Y, et al. SUMO E1 covalent allosteric inhibitors modulate polyamine synthesis via the MAT2A-AdoMetDC axis. 2024; 2024.12.12.627095

80. Ai Y, Xu S, Zhang Y, et al. High-efficiency discovery and structure-activity-relationship analysis of non-substrate-based covalent inhibitors of S-adenosylmethionine decarboxylase. 2024; 2024.09.07.611751

81. London N, Raveh B, Cohen E, et al. Rosetta FlexPepDock web server--high resolution modeling of peptide-protein interactions. Nucleic Acids Res 2011; 39:W249-253

82. Raveh B, London N, Schueler-Furman O. Sub-angstrom modeling of complexes between flexible peptides and globular proteins. Proteins 2010; 78:2029–2040

83. Tivon B, Gabizon R, Somsen BA, et al. Covalent flexible peptide docking in Rosetta. Chem Sci 2021; 12:10836–10847

84. HADDOCK developer team (2018). HADDOCK covalent docking tutorial. Available at: https://www.bonvinlab.org/education/biomolecular-simulations-2018/HADDOCK_tutorial/ (Accessed April 24th, 2023).

85. Honorato RV, Trellet ME, Jiménez-García B, et al. The HADDOCK2.4 web server for integrative modeling of biomolecular complexes. Nat Protoc 2024; 19:3219–3241

86. Mueller NPF, Carloni P, Alfonso-Prieto M. Molecular determinants of acrylamide neurotoxicity through covalent docking. Front Pharmacol 2023; 14:1125871

87. Anchoori RK, Jiang R, Peng S, et al. Covalent Rpn13-Binding Inhibitors for the Treatment of Ovarian Cancer. ACS Omega 2018; 3:11917–11929

88. Schulz R, Atef A, Becker D, et al. Phenylthiomethyl Ketone-Based Fragments Show Selective and Irreversible Inhibition of Enteroviral 3C Proteases. J Med Chem 2018; 61:1218–1230

89. Soubhye J, Chikh Alard I, Aldib I, et al. Discovery of Novel Potent Reversible and Irreversible Myeloperoxidase Inhibitors Using Virtual Screening Procedure. J Med Chem 2017; 60:6563–6586

90. Venugopala KN, Govender R, Khedr MA, et al. Design, synthesis, and computational studies on dihydropyrimidine scaffolds as potential lipoxygenase inhibitors and cancer chemopreventive agents. Drug Des Devel Ther 2015; 9:911–921

91. Irwin JJ, Shoichet BK. ZINC--a free database of commercially available compounds for virtual screening. J Chem Inf Model 2005; 45:177–182

92. Li G-B, Yang L-L, Wang W-J, et al. ID-Score: a new empirical scoring function based on a comprehensive set of descriptors related to protein-ligand interactions. J Chem Inf Model 2013; 53:592–600

93. Ehmke V, Winkler E, Banner DW, et al. Optimization of triazine nitriles as rhodesain inhibitors: structure-activity relationships, bioisosteric imidazopyridine nitriles, and X-ray crystal structure analysis with human cathepsin L. ChemMedChem 2013; 8:967–975

94. Hardegger LA, Kuhn B, Spinnler B, et al. Systematic investigation of halogen bonding in protein-ligand interactions. Angew Chem Int Ed Engl 2011; 50:314–318

95. Shenoy RT, Sivaraman J. Structural basis for reversible and irreversible inhibition of human cathepsin L by their respective dipeptidyl glyoxal and diazomethylketone inhibitors. J Struct Biol 2011; 173:14–19

96. Asaad N, Bethel PA, Coulson MD, et al. Dipeptidyl nitrile inhibitors of Cathepsin L. Bioorg Med Chem Lett 2009; 19:4280–4283

97. Daylight Theory: SMARTS - A Language for Describing Molecular Patterns. http://www.daylight.com/dayhtml/doc/theory/ theory.smarts.html.

98. Du J, Yan X, Liu Z, et al. cBinderDB: a covalent binding agent database. Bioinformatics 2017; 33:1258–1260

99. Du H, Gao J, Weng G, et al. CovalentInDB: a comprehensive database facilitating the discovery of covalent inhibitors. Nucleic Acids Res 2021; 49:D1122–D1129

100. Du H, Jiang D, Gao J, et al. Proteome-Wide Profiling of the Covalent-Druggable Cysteines with a Structure-Based Deep Graph Learning Network. Research (Wash D C) 2022; 2022:9873564

101. Zhang W, Pei J, Lai L. Statistical Analysis and Prediction of Covalent Ligand Targeted Cysteine Residues. J Chem Inf Model 2017; 57:1453–1460

102. Xu Y, Wang S, Hu Q, et al. CavityPlus: a web server for protein cavity detection with pharmacophore modelling, allosteric site identification and covalent ligand binding ability prediction. Nucleic Acids Research 2018; 46:W374–W379

103. Yu W, Weber DJ, MacKerell AD. An Integrated Covalent Drug Design Workflow using Site-Identification by Ligand Competitive Saturation. J Chem Theory Comput 2023; 19:3007–3021

104. Faller CE, Raman EP, MacKerell AD, et al. Site Identification by Ligand Competitive Saturation (SILCS) simulations for fragment-based drug design. Methods Mol Biol 2015; 1289:75–87

105. Lakkaraju SK, Raman EP, Yu W, et al. Sampling of Organic Solutes in Aqueous and Heterogeneous Environments Using Oscillating Excess Chemical Potentials in Grand Canonical-like Monte Carlo-Molecular Dynamics Simulations. J Chem Theory Comput 2014; 10:2281–2290

106. Backus KM, Correia BE, Lum KM, et al. Proteome-wide covalent ligand discovery in native biological systems. Nature 2016; 534:570–574

107. Soylu I, Marino SM. Cpipe: a comprehensive computational platform for sequence and structure-based analyses of Cysteine residues. Bioinformatics 2017; 33:2395–2396

108. Zhu K, Day T, Warshaviak D, et al. Antibody structure determination using a combination of homology modeling, energy-based refinement, and loop prediction. Proteins 2014; 82:1646–1655

109. Salam NK, Adzhigirey M, Sherman W, et al. Structure-based approach to the prediction of disulfide bonds in proteins. Protein Engineering, Design and Selection 2014; 27:365–374

110. Schrödinger Release 2025-1: BioLuminate, Schrödinger, LLC, New York, NY, 2025.

111. Gao M, Günther S. HyperCys: A Structure- and Sequence-Based Predictor of Hyper-Reactive Druggable Cysteines. Int J Mol Sci 2023; 24:5960

112. Knox C, Wilson M, Klinger CM, et al. DrugBank 6.0: the DrugBank Knowledgebase for 2024. Nucleic Acids Res 2024; 52:D1265–D1275

113. Liu T, Hwang L, Burley SK, et al. BindingDB in 2024: a FAIR knowledgebase of protein-small molecule binding data. Nucleic Acids Res 2025; 53:D1633–D1644

114. Gaulton A, Bellis LJ, Bento AP, et al. ChEMBL: a large-scale bioactivity database for drug discovery. Nucleic Acids Res 2012; 40:D1100–D1107

115. Kowalska D, Maculewicz J, Stepnowski P, et al. Interaction of pharmaceutical metabolites with blood proteins and membrane lipids in the view of bioconcentration: A preliminary study based on in vitro assessment. Science of The Total Environment 2021; 783:146987

116. Corsini A, Bortolini M. Drug-induced liver injury: the role of drug metabolism and transport. J Clin Pharmacol 2013; 53:463–474

117. Ye H, Nelson LJ, Gómez Del Moral M, et al. Dissecting the molecular pathophysiology of drug-induced liver injury. World J Gastroenterol 2018; 24:1373–1385

118. Abboud G, Kaplowitz N. Drug-induced liver injury. Drug Saf 2007; 30:277–294

119. Wu Y, Pan L, Chen Z, et al. Metabolite Identification in the Preclinical and Clinical Phase of Drug Development. Curr Drug Metab 2021; 22:838–857

120. Jee A, Sernoskie SC, Uetrecht J. Idiosyncratic Drug-Induced Liver Injury: Mechanistic and Clinical Challenges. Int J Mol Sci 2021; 22:2954

121. Stiborova M. Formation of Covalent DNA Adducts by Enzymatically Activated Carcinogens and Drugs In Vitro and Their Determination by 32P-postlabeling. J Vis Exp 2018; 57177

122. Kalgutkar AS, Didiuk MT. Structural alerts, reactive metabolites, and protein covalent binding: how reliable are these attributes as predictors of drug toxicity? Chem Biodivers 2009; 6:2115–2137

123. Zhou S, Chan E, Duan W, et al. Drug bioactivation, covalent binding to target proteins and toxicity relevance. Drug Metab Rev 2005; 37:41–213

124. Kalgutkar AS, Gardner I, Obach RS, et al. A comprehensive listing of bioactivation pathways of organic functional groups. Curr Drug Metab 2005; 6:161–225

125. Knowles SR, Uetrecht J, Shear NH. Idiosyncratic drug reactions: the reactive metabolite syndromes. Lancet 2000; 356:1587–1591

126. Srivastava A, Maggs JL, Antoine DJ, et al. Role of reactive metabolites in drug-induced hepatotoxicity. Handb Exp Pharmacol 2010; 165–194

127. Rendic SP, Guengerich FP. Formation of potentially toxic metabolites of drugs in reactions catalyzed by human drug-metabolizing enzymes. Arch Toxicol 2024; 98:1581–1628

128. Gillette JR, Lau SS, Monks TJ. Intra- and extra-cellular formation of metabolites from chemically reactive species. Biochem Soc Trans 1984; 12:4–7

129. Liu X, Lv H, Guo Y, et al. Structure-Based Reactivity Profiles of Reactive Metabolites with Glutathione. Chem Res Toxicol 2020; 33:1579–1593

130. Shenton JM, Chen J, Uetrecht JP. Animal models of idiosyncratic drug reactions. Chem Biol Interact 2004; 150:53–70

131. Dalvie D, Kalgutkar AS, Chen W. Practical approaches to resolving reactive metabolite liabilities in early discovery. Drug Metab Rev 2015; 47:56–70

132. Stepan AF, Walker DP, Bauman J, et al. Structural alert/reactive metabolite concept as applied in medicinal chemistry to mitigate the risk of idiosyncratic drug toxicity: a perspective based on the critical examination of trends in the top 200 drugs marketed in the United States. Chem Res Toxicol 2011; 24:1345–1410

133. Park BK, Boobis A, Clarke S, et al. Managing the challenge of chemically reactive metabolites in drug development. Nat Rev Drug Discov 2011; 10:292–306

134. Matlock MK, Hughes TB, Dahlin JL, et al. Modeling Small-Molecule Reactivity Identifies Promiscuous Bioactive Compounds. J Chem Inf Model 2018; 58:1483–1500

135. Niu H, Alvarez-Alvarez I, Chen M. Artificial Intelligence: An Emerging Tool for Studying Drug-Induced Liver Injury. Liver Int 2025; 45:e70038

136. Yang S, Kar S. Application of artificial intelligence and machine learning in early detection of adverse drug reactions (ADRs) and drug-induced toxicity. Artificial Intelligence Chemistry 2023; 1:100011

137. Kazmi SR, Jun R, Yu M-S, et al. In silico approaches and tools for the prediction of drug metabolism and fate: A review. Comput Biol Med 2019; 106:54–64

138. Wang D, Liu W, Shen Z, et al. Deep Learning Based Drug Metabolites Prediction. Front Pharmacol 2019; 10:1586

139. Litsa EE, Das P, Kavraki LE. Prediction of drug metabolites using neural machine translation. Chem Sci 2020; 11:12777–12788

140. Mazzolari A, Vistoli G, Testa B, et al. Prediction of the Formation of Reactive Metabolites by A Novel Classifier Approach Based on Enrichment Factor Optimization (EFO) as Implemented in the VEGA Program. Molecules 2018; 23:2955

141. Lin J, Li M, Mak W, et al. Applications of In Silico Models to Predict Drug-Induced Liver Injury. Toxics 2022; 10:788

142. Norman BH. Drug Induced Liver Injury (DILI). Mechanisms and Medicinal Chemistry Avoidance/Mitigation Strategies. J Med Chem 2020; 63:11397–11419

143. Di Zeo-Sánchez DE, Segovia-Zafra A, Matilla-Cabello G, et al. Modeling drug-induced liver injury: current status and future prospects. Expert Opin Drug Metab Toxicol 2022; 18:555–573
